# On the virtues and limitations of Granger-causal brain connectivity estimate: Critical analysis using neural mass models

**DOI:** 10.1162/NETN.a.38

**Published:** 2026-01-08

**Authors:** Silvana Pelle, Giulia Piermaria, Elisa Magosso, Mauro Ursino

**Affiliations:** Department of Electrical, Electronic and Information Engineering, Guglielmo Marconi, University of Bologna, Cesena, Italy

**Keywords:** Brain connectivity, Brain rhythms, Granger causality, Conditional estimation, Excitatory synapses, Inhibitory synapses

## Abstract

Estimation of brain connectivity from neuroelectric data is a fundamental problem in modern neuroscience, and it is used to assess the network properties of brain function. In the present work, we critically assess the virtues and limitations of temporal Granger causality (using both conditional and unconditional formulations) for the estimation of functional brain connectivity, using a neural mass model as the ground truth. The model simulates transmission among different brain rhythms (in the *θ*, *α*, *β*, and *γ* bands) via excitatory and inhibitory synapses. The results show that Granger causality is able to detect relative changes in connectivity, but the estimated values are influenced by the operative conditions (sampling frequency, signal length, delay). Moreover, the absolute value of Granger causality depends on the particular rhythm transmitted and is affected by nonlinear phenomena, especially the activity level in the connected regions. In the case of complex connectivity networks, conditional Granger causality overwhelms the unconditional one, since the latter often discovers spurious connections. Finally, inhibitory connections can be revealed more easily by Granger causality than similar excitatory connections, a result generally neglected in brain network studies. The present results can drive the correct interpretation of Granger-causality-based connectivity networks derived from neuroelectric signals.

## Author Summary

Granger causality is frequently used to assess brain connectivity from neuroimaging data. However, this method quantifies the influence of one signal on another, rather than detecting the underlying mechanisms. Here, we utilize a neurocomputational model capable of generating neuroelectric oscillatory signals with various frequencies to investigate the merits and limitations of G-causality in assessing underlying connectivity circuits. Results demonstrate that changes in connectivity can be detected; however, these are influenced by additional factors, including the frequency of the rhythms and nonlinear phenomena. Moreover, inhibitory connections have a greater impact on G-causality than excitatory connections. For complex networks comprising many nodes, a conditional approach, which takes all signals into account, is preferable to a unconditional one for statistically detecting or rejecting the presence of a connection.

## INTRODUCTION

The execution of perceptual, motor, or cognitive tasks in the brain requires the integrated participation of several specialized cerebral areas, reciprocally interconnected, which exchange information via complex neural circuits. Accordingly, there is an increasing interest in present neuroscience toward the characterization of the networks involved in specific tasks, their plastic behavior, and possible alterations in pathological conditions (Gaudet et al., 2020; Khaleghi et al., 2024; Power et al., 2010; van Diessen et al., 2015; van Mierlo et al., 2019).

Characterization of brain circuits is generally performed using connectivity estimation techniques, which aim to discover connections among data starting from neuroimaging signals (like fMRI, EEG, MEG, or intracortical recordings). These techniques, in turn, are usually divided into functional and effective connectivity: the first aims at describing the statistical dependencies among signals and discovering information that one signal transmits to another one, making only minimal assumptions about the underlying neural circuitry; conversely, the second aspires at finding the circuitry that can explain the observed signal characteristics (Cao et al., 2022; Friston, 2011; Li, 2023). Of course, the two methods provide different but complementary notions and are both useful in neuroscience.

Among the different methods for functional connectivity estimation, one of the most popular today is Granger causality (or briefly, G-causality or GC). Based on its original formulation (Granger, 1969), a time series X “Granger causes” another time series Y if, in a linear autoregressive (AR) model, the information contained in the past values of X improve the prediction of the future of Y beyond the information contained in the past values of Y and of any other variables in the system, with the improvement quantified in terms of the reduction of the prediction error variance. Thus, G-causality quantifies the portion of variability in a dependent variable (e.g., Y) that is accounted for specifically by one predictor variable (e.g., X), after controlling for the influence of the other predictors. This framework for functional connectivity estimation, in turn, can be applied both in the temporal and frequency domains (Geweke, 1982; Granger, 1969; Shahnazian et al., 2012). Although G-causality was criticized as severely biased or of high variance when compared with ground-truth AR models (Stokes & Purdon, 2017), these concerns have been promptly addressed and solved by various studies (Barnett et al., 2018; Dhamala et al., 2018; Faes et al., 2017). Today, G-causality is a widely used method to estimate brain functional connectivity in particular starting from high-density EEG signals joined with cortical source reconstruction, thanks to its ability to exploit the high temporal resolution of EEG without excessive computational costs, and to its capacity to provide directed connectivity measures in both the time and frequency domains (Bressler & Seth, 2011; Cekic et al., 2018; Koutlis et al., 2021; Seth et al., 2015; Sohrabpour et al., 2016).

Despite the interesting results recently obtained from G-causality in neuroscience, several problems are still debated and can benefit from additional investigation. In particular, the exact information obtainable from G-causality remains questionable, and the influence of several physiological and operative parameters on the obtained results still needs to be wholly assessed. Among the main problems deserving attention, we can mention:

i)  Many operative parameters can affect G-causality estimation, in particular, the order of the AR model, the signal length, and the sampling frequency of the signal.
ii)  Information transmission in the brain occurs via various brain rhythms characterized by different frequency bands. It is a common idea that brain oscillations and rhythm synchronization play an essential role in cognition (Cannon et al., 2014; Düzel et al., 2010; Fries, 2015; Klimesch et al., 2010; Vezoli et al., 2021). However, understanding the relationship between brain rhythm transmission and connectivity is still controversial. The same connectivity strength may allow sound rhythm transmission in some frequency bands but can be inadequate in other bands.
iii)  G-causality can be estimated either using unconditional or conditional methods (Barnett & Seth, 2014; Kaminski & Blinowska, 2022). In the second technique, all channels are taken into account in the AR model when estimating the interaction among any couple of signals (pairwise-conditional causality, as in the original definition of G-causality). In contrast, in the first method, the analysis of each signal pair is conducted separately (pairwise-unconditional causality). Unconditional estimation generally produces more stable estimates due to the smaller number of parameters involved. Still, it may have problems in accounting for indirect interactions, such as those due to a common input (Bastos & Schoffelen, 2016; Chiarion et al., 2023).
iv)  Nonlinearities play a fundamental role in neural dynamics; conversely, the classic Granger approach uses a linear AR model. The way nonlinear terms (such as the presence of a threshold or saturation) affect the capacity to transmit information from one neural population to another, hence modulating functional connectivity, deserves a quantitative investigation.
v)  Finally, interactions among neural populations are not only excitatory but also inhibitory in type. Of course, G-causality is unable to distinguish between an excitatory and an inhibitory connection. We are aware that no previous study has investigated the different consequences that excitation or inhibition can have on G-causality estimates within a complex connectivity network.

The previous questions can be assessed using neurocomputational models inspired by brain functioning, in which a well-known connectivity structure is imposed among neural populations to generate surrogate signals. Among the different models available, the so-called neural mass models summarize the dynamics of a whole region using a limited number of state variables (Cona et al., 2011; David & Friston, 2003; Jansen & Rit, 1995; Moran et al., 2007; Ursino et al., 2010; Zavaglia et al., 2006). These models mimic entire populations of synchronized neurons and are particularly suitable for generating different rhythms and mimicking their propagation among brain areas.

The present study aims to quantitatively analyze the previous controversial points by applying temporal G-causality to simulated signals obtained via a biologically inspired neural mass model of interconnected brain regions. The connectivity values imposed on the model (or alternatively the generated postsynaptic currents) are used as the ground truth and compared with the G-causality values estimated using unconditional or conditional methods.

As is well known, G-causality is designed to study effects, not the underlying mechanisms, and G-causality is not supposed to recapitulate changes in a physical generative model (Bressler & Seth, 2011; Seth et al., 2015). In order to disclose generative mechanisms from signal measurements, other techniques based on an underlying model (such as dynamic causal modeling) are more appropriate (Cao et al., 2022; Daunizeau et al., 2011; Friston, 2011; Friston et al., 2003). Accordingly, we do not expect that G-causality estimates to depend linearly on synapse weight; rather, we guess that the relationship between G-causality estimate and synaptic weight fails in many circumstances, and can also provide counterintuitive results. However, since G-causality is frequently used in neuroscience to derive information on brain connectivity, we found it of value to analyze in which conditions one can infer a relationship between changes in G-causality and the underlying physical mechanism, and in which conditions this relationship is blurred or broken. This may be of value in giving some indications that help the interpretation of changes in connectivity graphs obtained from G-causality, and help avoid errors and misunderstanding.

## RESULTS

We simulated an imposed connectivity pattern between two or more brain regions characterized by distinct intrinsic rhythms, and estimated the temporal G-causality from the time series of the simulated regions. G-causality between pair of time series was computed in MATLAB using both an unconditional approach (see Equation 1 in Granger Causality (G-causality) section) as implemented by the Brainstorm (BS) toolbox and a conditional approach (see Equation 2 in Granger Causality (G-causality) section) as implemented by the MVCG toolbox. In the following, the term *BS G-causality* will be used to denote the unconditional estimates obtained using the BS software, and the term *MVGC G-causality* will be used to denote the estimates obtained using the MVCG software. Note that as a comparison, in the case of three or more signals, the MVGC toolbox is used to compute both conditional and unconditional G-causality. As BS and MVGC are two popular toolboxes, we deemed it interesting to evidence their convergence or divergence and the different behavior between the conditional and unconditional approaches.

The simulations described in the following sections were performed using a signal length of 10 s, an AR model order of 15 (estimated from the Akaike criterion), a connectivity temporal delay of 10 ms, and a sampling frequency of 100 Hz.

### Interaction Between Two Rhythms

The first set of simulations assumes two different regions producing two distinct rhythms and connected via reciprocal feedback excitatory connections. We evaluated the effect of a progressive increase in one connection on G-causality and power spectrum density while maintaining the other connection at a constant intermediate value.

**Figure 1.**
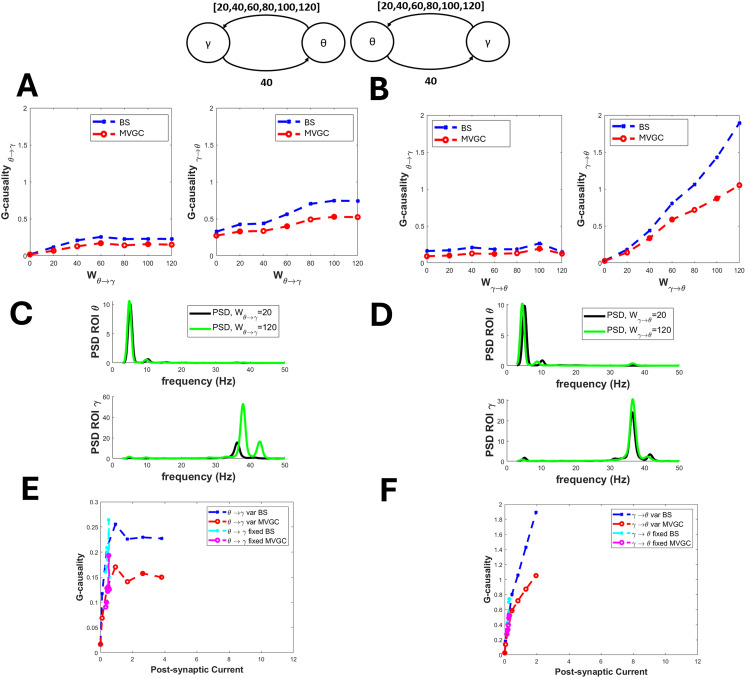
Effect of a connectivity change on the causal transmission between a gamma and a theta rhythm. G-causality was estimated using both the BS toolbox (denoted by “BS”) and the MVCG toolbox (denoted by “MVGC”). (A) refers to a progressive increase in connectivity from the theta to the gamma region (from 0 to 120), while the connectivity from gamma to theta is fixed at 40. In this condition, the G-causality from theta to gamma increases moderately, and that from gamma to theta increases too. Power spectral density (C) exhibits a large increase in the gamma region. (B) refers to a progressive increase in the connectivity from the gamma to theta region (from 0 to 120), while the connectivity from theta to gamma is fixed at 40. In this condition, G-causality from gamma to theta increases dramatically, whereas that from theta to gamma is almost unaffected. This is also reflected in the power spectral density of the two regions (D), which shows the appearance of a gamma rhythm in the theta region. Finally, (E) and (F) show the relationship between postsynaptic current and G-causality, evaluated when the connection is progressively increasing (denoted “var”) or when the connection is fixed and the postsynaptic current varies as a consequence of an increase in the opposite re-entrant connection (denoted “fixed”).

Figure 1 shows the effects of some changes in the theta-gamma connection. As a general rule (always verified in all the following figures), MVGC G-causality exhibits smaller values than the BS one. An increase in the connection from the gamma region toward the theta region produces a considerable increase in gamma-to-theta G-causality without any influence on the other (fixed) one (Figure 1B). In particular, if *γ* → *θ* connectivity increases from 20 to 120, the BS G-causality increases from 0.2 to 2.0, which represents a disproportionate rise. Interestingly, the increase in MVGC G-causality is less evident (from approximately 0.15 to 1), which is much more proportional to the actual percentage increase imposed in the model. On the contrary, an increase in the connectivity *θ* → *γ* produces only minor effects (Figure 1A). Strangely, high values of the *θ* → *γ* connectivity (above 60) produce an “apparent” increase in G-causality in the opposite direction. We can conclude that the gamma rhythm produces a high G-causality toward the theta one, whereas the opposite transmission is much weaker. These alterations are also reflected in the power spectral densities (Figures 1C and 1D). The last shows that an increase in the *γ* → *θ* connectivity produces a slightly higher spectrum both in the gamma and the theta bands, with the appearance of gamma power in the theta region, which receives the rhythm from the other. An increase in the connectivity *θ* → *γ* produces a power increase in the gamma region (in the gamma band) with only minor changes in the theta region.

**Figure 2.**
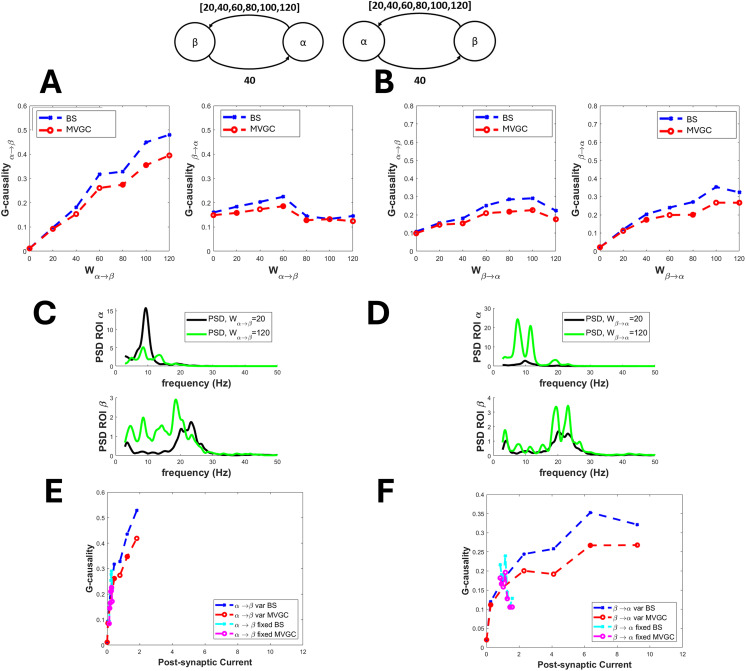
Effect of a connectivity change on the causal transmission between an alpha and a beta rhythm. G-causality was estimated using both the BS toolbox (denoted by “BS”) and the MVCG toolbox (denoted by “MVGC”). (A) refers to a progressive increase in connectivity from the alpha to the beta region (from 0 to 120), while the connectivity from beta to alpha is fixed at 40. In this condition, G-causality from alpha to beta increases dramatically, whereas that from beta to alpha first increases and then decreases. This is also reflected in the power spectral density of the two regions (C). (B) refers to a progressive increase in connectivity from the beta to the alpha region (from 0 to 120). In this condition, G-causality increases moderately in both directions, and both PSD spectra also increase (D). Finally, (E) and (F) show the relationship between postsynaptic current and GC, evaluated when the connection is progressively increasing (denoted “var”) or when the connection is fixed and the postsynaptic current varies as a consequence of an increase in the opposite re-entrant connection (denoted “fixed”).

The connection between the alpha and beta rhythms is summarized in Figure 2. The transmission from alpha to beta can be reproduced quite well by the BS G-causality estimator, which exhibits approximately a fivefold increase when *α* → *β* connectivity increases from 20 to 120. In contrast, the G-causality estimate in the opposite direction remains pretty constant or even moderately decreases. Interestingly, in this case, the MVGC G-causality estimator is less sensitive. Conversely, the transmission from beta to alpha is weaker (G-causality exhibits just a threefold increase), and an “apparent” increase becomes evident in the opposite direction (*α* → *β* G-causality; Figure 2B). As to frequency spectra, strangely, an increase in the *α* → *β* connectivity produces a decrease in the alpha power in the *α* region and a significant power increase in the overall frequency band 5–20 Hz in the *β* region. An increase in the *β* → *α* connectivity, in turn, increases the power spectrum in both regions, with the appearance of some beta power in the *α* region.

The effects of connectivity changes between alpha and theta regions, gamma and beta regions, and gamma and alpha regions are shown in Supporting Information S2 (Supporting Information Figures S2, S3, and S4). These figures confirm that (a) the gamma rhythm can be transmitted strongly, but it requires high connectivity; (b) the alpha rhythm transmission increases proportionally to the connectivity strength; (c) the transmission of the beta rhythm is weaker; and (d) theta rhythm G-causality is small and scarcely affected by the connectivity values.

Finally, in all previous figures, we also report the relationship between G-causality and the postsynaptic current, since this is the primary effect of the synapse. Results show that this relationship, when evaluated using the synapse that is actually varying, resembles the relationship between GC versus synaptic strength, with an approximately linear trend, and possibly exhibiting upper saturation. In this case too, the slope of this relationship is strongly affected by the frequency of the presynaptic and postsynaptic rhythms (for instance, it is much higher considering the *γ* → *θ* and *γ* → *β* relationships, and smaller for the *θ* → *γ* and *θ* → *α* relationships). The same relationship, evaluated when varying the other re-entrant connection, exhibits similar values, but is much noisier due to the smaller changes in postsynaptic current induced by the secondary effect of the opposite synapse, which make a correct evaluation difficult.

A sensitivity analysis on some parameters (signal length, AR model order, temporal delay, and sampling frequency) is reported in Supporting Information Figure S5 of the Supporting Information S2. Results are expected and can be summarized as follows: (a) the use of a high sampling frequency does not add information. Still, a higher model order is required to fit the time delay. Hence, a good suggestion can be to maintain sampling frequency just at a minimum (above twice the Nyquist frequency); (b) model order should reflect a balance between the need for accurate fitting (which requires enough parameters) and the reduction in statistical uncertainty (which increases with the parameters). In this work, we used the Akaike criterion for all simulations and found values ranging between 9 and 15 depending on the particular model. So, a value as high as 15 was used for all basic simulations to allow for a direct comparison. However, a model order as low as 8–9 is generally suitable, as shown in Supporting Information Figure S5 of the Supporting Information S2; (c) the signal length should be at least 2–3 s, and a longer length does not improve the results.

### Effect of the External Input to a Region

**Figure 3.**
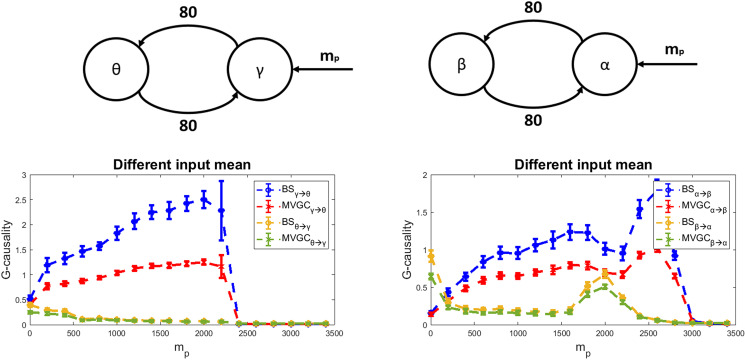
Effect of the input mean value to pyramidal neurons on G-causality estimation. The simulations were performed using two regions reciprocally interconnected (*θ* − *γ*: left panels, or *α* − *β*: right panels), with both connectivity values as high as 80. We assumed that the input to pyramidal neurons in the left region has a mean value *m*_*p*_ set to 400. In contrast, the mean value *m*_*p*_ of the input to pyramidal neurons of the right region is progressively increased from 0 (no excitation) to +3500 (strong excitation). G-causality was estimated using both the BS toolbox (blue and brown lines denoted by “BS”) and MVCG toolbox (red and green lines denoted by “MVGC”). Results are provided as mean + std over 20 trials. G-causality turns out very small at small input values, then progressively increases. At high input values, G-causality abruptly falls to zero due to saturation. G-causality in the opposite direction exhibits some “apparent” changes in case of the *α* − *β* regions.

Nonlinearities can significantly affect the behavior of neural populations, basically due to the presence of a lower threshold and upper saturation. Figure 3 explores the effect of a change in the input to a gamma region (left) or a change in the input to the alpha population (right) on the estimated G-causality in the same cases as Figures 1 and 2 (i.e., a gamma-theta coupling and an alpha-beta coupling). The basic idea is that a change in the input modifies the working point on a nonlinear curve, thus affecting information transmission. Indeed, both at low and high input values, the estimated G-causality falls. The reason is that the presynaptic population is almost wholly inhibited or works in a saturation condition (hence with almost constant upper frequency) and hence does not transmit temporal information. Moreover, while the upper saturation state produces quite a sharp fall in G-causality, increasing the input from low values produces a gradual rise in G-causality. Interestingly, the increase in G-causality in one direction is generally paralleled by a decrease in the opposite direction (from theta to gamma or from beta to alpha). Finally, it is worth noting the complex alterations occurring in the alpha-beta causality at high values of the input (*m*_*p*_ ≃ 2000), characterized by a sudden “apparent” increase in the causality from beta to alpha. The previous results suggest that the connectivity networks obtained from functional connectivity estimates reflect not only the authentic connections among populations but also the excitation levels that are consequent on external inputs.

### The Effect of a Common Input

**Figure 4.**
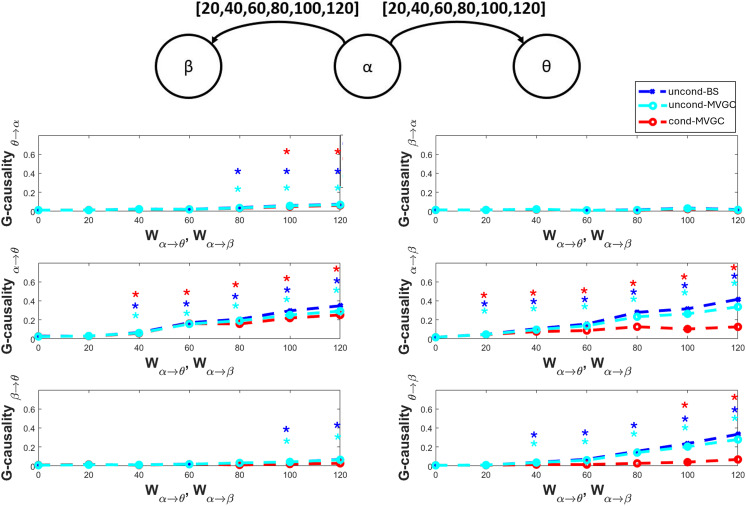
Effect of a common input on G-causality estimation. The common input originates from an *α* region and targets both a *θ* region and a *β* region with the same connectivity strength. This connectivity is progressively increased from 20 to 120 (as indicated in the upper panel). The other panels represent the G-causality estimate of all six possible connections. G-causality was estimated using both the unconditional BS toolbox (blue lines denoted by “uncond-BS”) and the MVCG toolbox (unconditional formulation, cyan lines denoted with “uncond-MVGC,” and conditional formulation, red lines denoted by “cond-MVGC”). Asterisks denote statistically significant G-causality at 5% (blue, cyan, or red, as in the corresponding plots). All *α* → *β* and *α* → *θ* G-causality estimates, corresponding to existing “physical” connections, are statistically significant (except in only one case for *α* → *θ*) and exhibit a progressive increase. A feedback connection *θ* → *α* is detected by all estimators at high connectivity values. False connectivity *β* → *θ* and *θ* → *β*, caused by the common input, are especially detected by the unconditional estimators, whereas the conditional estimator is more robust against this spurious effect.

The previous simulations, which involve only two interacting regions, do not show significant differences between the BS G-causality estimate and the MVGC G-causality estimate (apart from the basal value, which is higher for the first). Different behaviors between the two estimators may emerge more clearly when more than two regions are involved. In particular, errors in functional connectivity estimates can arise in the presence of a common input to two nonconnected populations, coming from a third population. In this situation, the two populations can be seen as erroneously connected since the fluctuations in the common input are reflected in both time series, producing a spurious correlation; hence, it is expected that conditional estimates outperform the unconditional ones. This problem is analyzed in Figure 4 when we simulate the presence of a common input coming from an alpha region, entering into two regions with theta and beta rhythms, respectively. The common connectivity from the alpha region is then increased from 20 to 120. All the six different possible connectivity values are estimated (not only the “physical” connectivities *α* → *θ* and *α* → *β*, but also the remaining four ones) from the three simulated signals during 20 trials, and a statistical significance test is performed on the obtained G-causality data (see Statistical Inference section). The results show that both the unconditional and conditional estimators follow the increase in the “physical” connectivity values. When the connectivity strength is small (W_*α*→*θ*_ = W_*α*→*β*_ = 20), the significance from alpha to theta is lost. When the connectivity value becomes higher or equal to 80, a spurious feedback connection from theta to alpha becomes statistically significant for both unconditional estimators (≥100 for the conditional estimator). More importantly, when the common connectivity strength is greater or equal to 40, the unconditional estimator also feels significant spurious connectivity from theta to beta. When connectivity becomes 100, unconditional G-causality also feels connectivity from beta to theta. Only when the connectivity strength reaches the value of 100 does the conditional estimator find a significant spurious connection from theta to beta. In conclusion, the presence of a common input can induce spurious connectivity between two nonconnected regions, and the conditional estimator is less prone to these errors. A similar result concerning a common gamma rhythm transmitted to a theta and a beta region is reported in Supporting Information Figure S6 of Supporting Information S2.

### A Brain Network With Excitatory Connections

**Figure 5.**
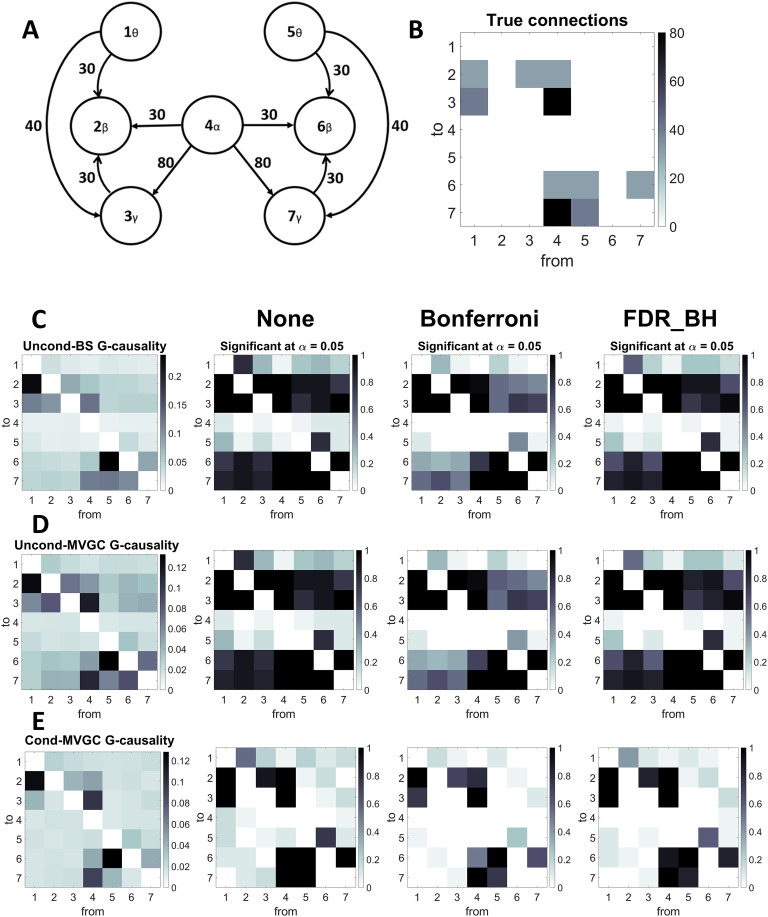
G-causality in case of a brain-inspired network with seven ROIs: two prefrontal (left and right) working in theta, two motors (left and right) working in beta, two occipital (left and right) in gamma, and one (thalamic) in alpha. In this simulation, we assumed that only excitatory connections are present (A). (B) represents the value of the true connections between regions. The columns in the matrix represent the source region, and the rows represent the target region (hence, the element in position i, j represents the connectivity W_i,j_ from region j to region i). (C)–(E) show results from G-causality estimation (unconditional BS, unconditional MVCG and conditional MVGC, respectively). In particular, these panels report the values obtained by G-causality estimation (mean values over 20 trials) and the fraction of trials in which a connection has been found statistically significant with alpha = 0.05 (with no correction, Bonferroni correction or FDR correction).

The previous results were concerned with either two interconnected populations or two nonconnected populations receiving a common input signal. Figure 5 shows the result of simulations performed using a more complex brain-inspired circuit, in which all four rhythms are present together and linked via excitatory connections. In particular, we assumed two frontal theta regions (left and right), two motor beta regions (left and right), two occipital gamma regions (left and right), and a central region (maybe thalamic) generating the alpha rhythm. Twenty simulations were performed on this circuit, and we estimated all 42 possible G-causality values. Moreover, we evaluated the number of cases in which a connection resulted statistically significant using the unconditional (both BS and MVGC) and conditional (MVGC) formulation. The significance test was either uncorrected or corrected with the Bonferroni correction or the false discovery rate (FDR) correction (Benjamini-Hochberg procedure). Results show that the conditional G-causality can discover the correct network quite well, mainly if a correction is performed. In particular, with the FDR correction, conditional G-causality finds the existence of correct connectivity in almost 100% of cases (true positives); however, some spurious connections (false positives) are sometimes found, especially from Region 6 to Region 5 and from Region 2 to Region 1 (both connections are from beta to theta). The use of Bonferroni correction is more stringent, resulting in a smaller number of spurious connections and some failures to discover the correct ones. Conversely, both unconditional G-causality find a lot of spurious connections, in particular, those entering into the beta and gamma regions.

### The Role of Inhibitory Connections

Although our neural mass model assumes that interregion connections are only mediated through long-range excitatory glutamatergic synapses originating from pyramidal neurons, one region can inhibit another region via disynaptic connections, from pyramidal neurons in the presynaptic region to postsynaptic fast inhibitory interneurons in the postsynaptic region (see Supporting Information S1). Of course, G-causality is unable to discriminate between excitatory and disynaptic inhibitory connections.

**Figure 6.**
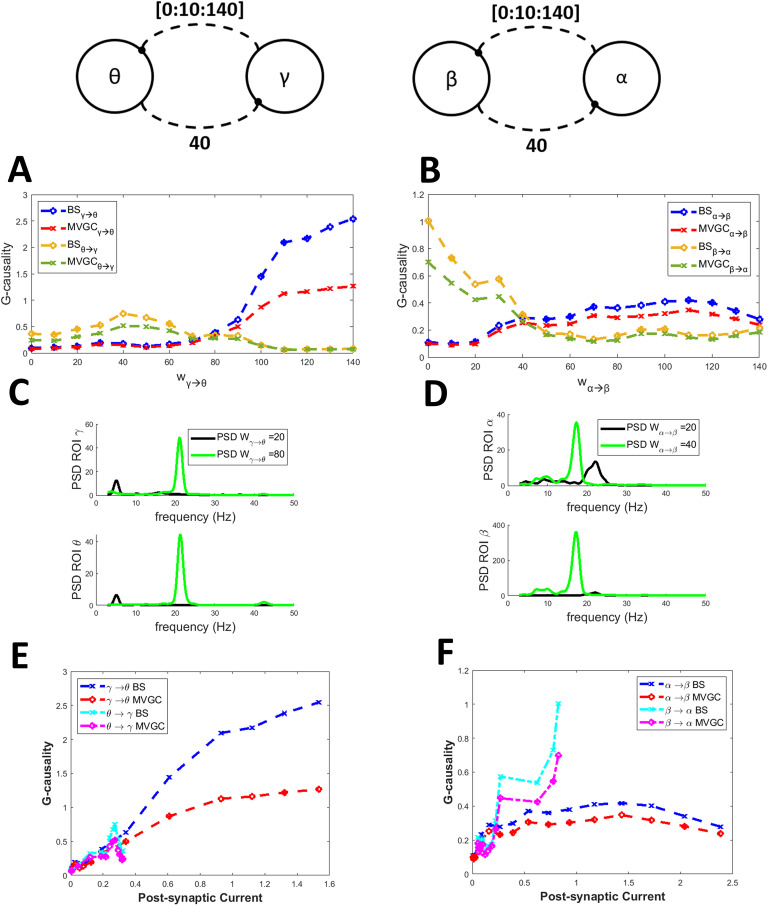
Effect of the change of an inhibitory connection on G-causality estimate. The simulations were performed using two regions with different rhythms (*θ* − *γ*: left panels, *α* − *β*: right panels) coupled via two inhibitory connections (as indicated in the upper panels). (A, B) refer to a progressive increase in one inhibitory connection (from 0 to 140), while the other connection is kept constant. G-causality was estimated using both the BS toolbox (blue and orange lines denoted by “BS”) and the MVCG toolbox (red and green lines denoted by “MVGC”) It is worth noting that the effect of inhibitory synapses on G-causality is stronger than the effect of excitatory synapses (compared with Figures 1 and 2). (C, D) show the power spectral densities. The presence of a strong inhibitory connection is able to produce a clear rhythm in the target region. (E, F) show the relationships of G-causality versus postsynaptic current in all cases.

Figure 6 simulates the connections between two regions arranged in feedback (*γ* − *θ* and *α* − *β*), when both connections are inhibitory. We then assumed that one inhibitory connection progressively increases. In this condition, the progressive increase in one connection produces a well-evident increase in the associated G-causality estimate, but this is paralleled by a progressive decrease in the other connection. Interestingly, G-causality seems more affected by inhibitory connections than by excitatory ones (let us compare the value of the inhibitory G-causality in Figure 6 with the excitatory values in Figures 1 and 2). Moreover, the *γ* → *θ* G-causality is much higher than the *α* → *β* one. Furthermore and accordingly, looking at the power spectral densities in the central panels of Figure 6, the presence of a strong inhibitory connection appears extremely powerful in transmitting a rhythm from the upstream to the downstream region. Finally, the relationship G-causality versus postsynaptic current appears overall increasing in all cases (at variance with G-causality vs. synaptic strength).

An example in which the increasing connection is inhibitory but the other (fixed) connection is excitatory is shown in the Supporting Information S2 (Supporting Information Figure S7). In this case, the excitatory G-causality remains quite constant while the other (inhibitory) is increasing.

**Figure 7.**
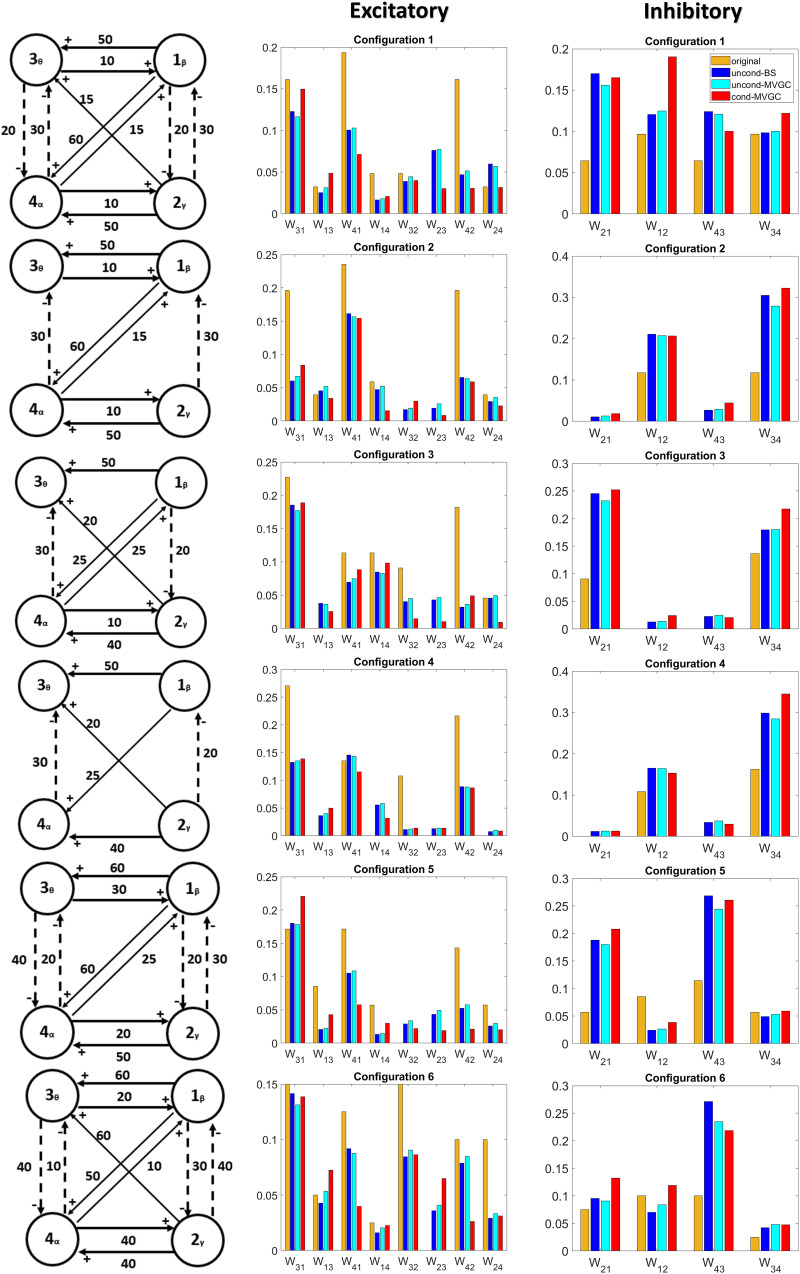
Estimation of G-causality in six different simulations, each performed with four interconnected ROIs, including excitatory and inhibitory connections. Each row refers to a different network configuration. The first column shows the network configuration (continuous lines denote excitatory connections, dashed lines inhibitory connections). The second column compares the values of the excitatory connections in the model with the unconditional (BS and MVGC) and conditional (MVCG) G-causality estimates; the third column compares the values of the inhibitory connections. To allow a comparison, the sum of all connections obtained with a given method is normalized to one. It is evident that inhibitory connections are overestimated, whereas excitatory connections are slightly underestimated by G-causality.

In order to better unmask the previous result and analyze the capacity of G-causality to reproduce both excitatory and inhibitory synapses in the same network, we realized several networks in which the four rhythms interact together via a combination of excitatory and inhibitory links. Six examples of such networks are shown in Figure 7. The six circuit arrangements are shown in the left column. The middle column represents the excitatory synapses via a bar graph: the actual values used in the model are in yellow; the G-causality values estimated with BS (uncond-BS), and with MVGC, both unconditional (uncond-MVGC) and conditional (cond-MVGC), are in blue, cyan, and red, respectively. The right column represents the inhibitory synapses with the same color code. To allow a direct comparison, all these values are normalized (i.e., the sum of all excitatory + inhibitory synapses evaluated with the same technique is equal to 1). As it is evident from this figure, G-causality tends to underestimate the value of excitatory connections but widely overestimates the value of inhibitory ones. Spurious synapses are often found by G-causality, but they are usually small compared with the actual ones. This result confirms the tendency of G-causality to overestimate inhibitory links compared with the excitatory ones.

### A Brain Network With Inhibitory Connections

**Figure 8.**
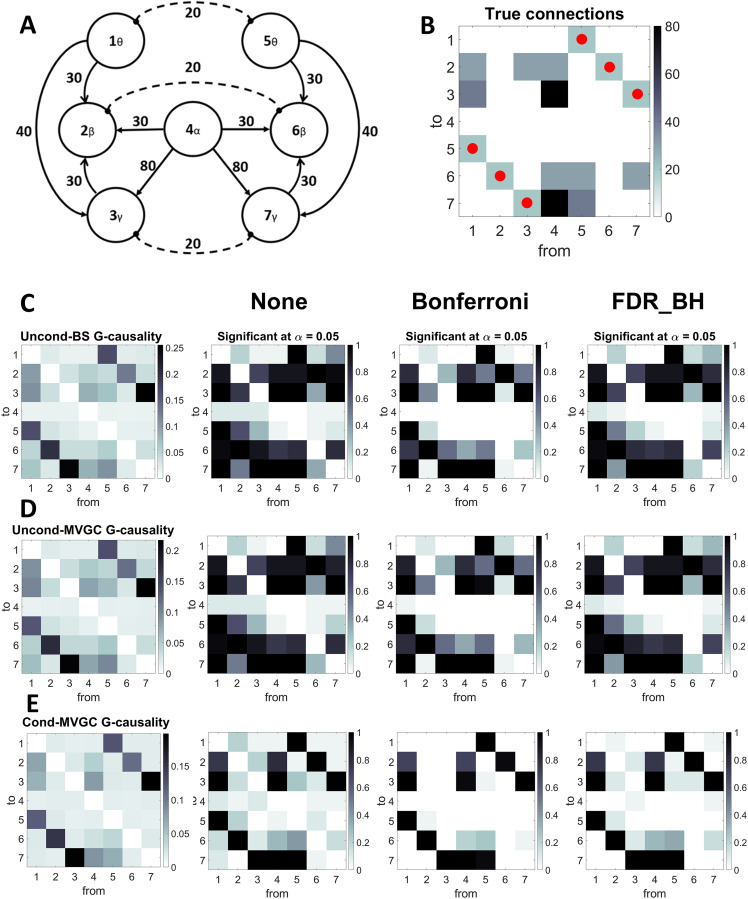
G-causality in case of a brain-inspired network with seven ROIs: two prefrontal (left and right) working in theta, two motors (left and right) working in beta, two occipital (left and right) in gamma, and one (thalamic) in alpha. In this simulation, we assumed that the analogous regions in the left and right hemispheres are linked by inhibitory connections (A). (B) represents the value of the true connections between regions. The inhibitory connections are marked with a red circle. The columns in the matrix represent the source region; rows represent the target region (hence, the element in position i, j represents the connectivity W_i,j_ from region j to region i). (C)–(E) show results from G-causality estimation (unconditional BS, unconditional MVGC, and conditional MVCG respectively). In particular, these panels report the values obtained by G-causality estimation (mean values over 20 trials) and the fraction of trials in which a connection has been found statistically significant with alpha = 0.05 (with no correction, Bonferroni correction, or FDR correction).

Finally, we repeated the same simulations as in Figure 5. Now the simplified brain-inspired network includes both excitatory and inhibitory (disynaptic) connections (see Figure 8). The difference compared with Figure 5 is that we now consider the presence of inhibitory links among analogous populations located in the left and right hemisphere, in accordance with the inhibitory theory of meta control proposed by Banich (i.e., information presented to both hemispheres is entirely managed by one dominant hemisphere [Banich, 1998; Kimura, 1972], which tries to inhibit the activity of the opposing hemisphere). Of course, this is just one possible example; other inhibitory-excitatory networks can be designed. Results confirm that the conditional G-causality outperforms the unconditional one, especially if the statistical test is corrected. The six inhibitory connections are always correctly detected (100% of corrected statistical results). In contrast, some excitatory connections are sometimes lost (for instance, from Region 3 to Region 2 and from Region 7 to Region 6, i.e., from gamma to beta; also, the connections from Regions 4 or 5 to Region 6 are frequently lost if statistical correction is implemented). Some spurious connections are also rarely found. As in the previous case, the unconditional G-causality estimates detect significant spurious connections in a majority of cases. This result confirms the superiority of the conditional estimator in the case of complex nets and the more robust detection of inhibitory links compared with the excitatory ones.

## DISCUSSION

This work aimed to assess the reliability and significance of temporal G-causality, using a neural mass model of interconnected regions of interest (ROIs) as a ground truth. In particular, we evaluated the problem of information transmission between different rhythms, considering the pivotal role that brain oscillations play in many cognitive problems. Importantly, we evaluated both excitatory and inhibitory connectivity, given the fundamental importance of inhibition in brain functioning. Moreover, we compared the results obtained with two different methods, unconditional and conditional, implemented by different toolboxes (BS only unconditional, and MVGC both unconditional and conditional). This comparison adds complexity to the results; however, since both methods and both toolboxes are widely used in the literature, it enhances the completeness of our analysis and provides indications on which estimator should be preferred in a given condition.

We are aware that Granger is not oriented to a generative model (Barrett & Barnett, 2013). Still, we thought it would be of value for people working with G-causality to investigate possible relationships and divergence between this technique and the underlying mechanisms. To avoid any misunderstanding, we do not affirm that G-causality is strictly related to model parameters. In fact, many results in this manuscript suggest that this relationship is frequently broken. Furthermore, nonlinear phenomena clearly alter this relationship, and even when a linear relationship is evident, it can be quite different depending on the rhythms involved. More simply, we show that, in several circumstances, inference about changes (rather than absolute values) in the underlying synaptic weights can be obtained from G-causality. Moreover, conditional MVGC provides relatively reliable statistical information on the presence or absence of a connection. On the other hand, we also provide evidence that in several cases, Granger connectivity provides counterintuitive indications.

In particular, our results confirm that the coupling strength is not the unique factor responsible for G-causality values. Still, several other conditions can mask the relationship between the coupling strength and G-causality or make this relationship widely variable. For instance, a very high input (Figure 3) or a high coupling factor can move a region to saturation, thus reducing the information transmitted from the source region or the predictability of the target signal. More generally, the working point of a population significantly affects G-causality due to the intrinsic nonlinear characteristics of neurons. Furthermore, different rhythms can be transmitted more effectively or less effectively, depending on frequency and synapse dynamics.

### Changes in Excitatory Connection

In the first portion of our study, we assumed the presence of excitatory connections between two regions. We evaluated the capacity of G-causality estimators to detect the progressive increase in one connection. Results confirm that a continuous rise in one connectivity is reflected in a continuous rise in G-causality; however, this latter is not proportional to the true connectivity change and can differ dramatically depending on the particular rhythm being transmitted.

Since connectivity cannot be separated among the different bands due to nonlinear interference, this work employs temporal G-causality, which encompasses the overall signal content and does not separate the bands. In particular, we never limit our analysis to a specific frequency range. Furthermore, in the case of multiple interconnected regions (as seen in the simulations of Figures 5, 7, and 8), various rhythms are superimposed in each region, collectively occupying a broad band. At the same time, nonlinearity produces intermodulation among the different frequencies. Consequently, we do not treat the individual bands or frequencies separately and do not employ a frequency-based approach. However, we assumed that each region exhibits an intrinsic rhythm when isolated from the others, and we studied how the rhythm in one region can affect the activity in another region, as well as whether this rhythm influence can be detected using G-causality. Our results indicate that the frequency of a rhythm significantly affects G-causality estimates; in particular, the theta rhythm transmission is not easily detectable with GC, whereas the transmission of a gamma rhythm has a substantial impact on G-causality estimates. This result agrees with the observation (Stokes & Purdon, 2017) that G-causality is primarily sensitive to the transmission of moment-to-moment variations and is inherently less sensitive to low-frequency.

Interestingly, by simulating a sixfold increase in one connection (from 20 to 120), we found quite a linear increase in G-causality concerning the transmission of alpha to beta and a mild increase concerning beta. G-causality from gamma can assume higher values but increases especially at high connectivity. A comparison between the BS and MVGC estimators suggests that both underestimate the percentage increase in beta connectivity, and both provide reliable percentage changes in alpha connectivity. In contrast, the BS estimator strongly overestimates the percentage variations in gamma connectivity.

Another interesting result is that, after an increase in one connectivity within a feedback circuit, the other re-entrant connectivity (assumed as constant) can exhibit an “apparent” change. We found a moderate “apparent” increase concerning the alpha and gamma rhythms and a moderate “apparent” decrease as to beta, while almost no change was detected concerning the theta connectivity.

The main conclusion of this preliminary study is that the sensitivity of G-causality can be different depending on which rhythm is transmitted from the presynaptic region to the postsynaptic one. Hence, there is no unique interpretation that is valuable for all rhythms.

### The Level of External Excitation

Nonlinearities are a typical characteristic of neural systems, mainly related to the presence of threshold and saturation for neural activities. Classic G-causality estimators, however, resort to linear AR models to predict future signals from past values. We tested the effect of nonlinearity by progressively changing the mean value of the external excitatory input to one ROI in the case of two regions connected in feedback. Simulations show that when the external input is either too small or too large, the G-causality estimation falls dramatically despite an intact connectivity strength. In our opinion, the reason is that G-causality mainly detects how much information one region is able to transmit to another one, affecting its temporal behavior. Suppose the presynaptic region is almost silent (due to a minimal excitatory input) or is working close to saturation (due to a very high excitatory input). In that case, the information transmitted is almost constant; hence, it is unable to help predict future values of the target region. Interestingly, as evident in Figure 3, changing the excitatory input to one region not only affects the causal link from this region to the other but also causes an “apparent” change in the reciprocal connection, although in a complex way.

### Effect of a Common Input

In all previous cases, we detected only minor differences between the G-causality estimators based on the BS and MVGC algorithms. Indeed, these simulations regarded just two regions connected in feedback. We expect substantial differences between the unconditional and conditional approaches when more than two regions are involved.

The first case concerns the presence of a common time-varying input from one region, reaching two other regions not reciprocally connected. This case clearly reveals the superiority of the conditional G-causality estimate: the conditional MVGC algorithm appears much more robust against the presence of “spurious” connections. If the common input is progressively increased, unconditional G-causality (both with BS and MVGC) finds a statistically significant connection between two unconnected regions, even at moderate values of the common input. In contrast, only at high values of the common input, the conditional estimator fails.

### Several Interconnected Regions

Comparison between the unconditional and conditional estimations becomes even more evident when dealing with complex nets involving several regions with multiple connections. In particular, we simulated a simplified brain-inspired circuit with seven ROIs, which exchange all rhythms together according to a physiological schema (two frontal regions in theta, two motor regions in beta, two sensory gamma regions, and one control-thalamic alpha region). The results confirm the superiority of the conditional estimate, suggesting that a conditional estimation, together with the statistical *F* test with Bonferroni or FDR correction, ensures a good compromise between sensitivity (identification of true positives) and specificity (identification of true negatives). Conversely, the unconditional estimates find a plethora of false positives.

### Analysis of Inhibitory Connections

An essential new aspect of this work concerns the detection of possible inhibitory connections. We are not aware of previous studies that have analyzed this aspect in the field of functional connectivity. Two premises must be stated before our analysis. First, inhibitory connectivity in our model has been described assuming that long-range connections originate from pyramidal neurons in the source region (hence, are only glutamatergic-excitatory in type) and target fast GABAergic (inhibitory) interneurons in the postsynaptic region. Future studies may consider more complex connectivity, for instance, by incorporating additional layers within the cortical column. Second, G-causality is always positive, and so it is intrinsically unable to distinguish between an excitatory and an inhibitory connectivity. Just the strength of this connection can be estimated without a sign.

Our simulations provide a new and perhaps unexpected result. Not only is G-causality able to detect a progressive change in inhibitory connectivity but it is also more sensitive to inhibitory than to excitatory connections. This is evident both using two interconnected ROIs (Figure 6 and Supporting Information S2) and more complex circuits involving four rhythms with various arrangements of excitatory and inhibitory connections (Figure 7). In the latter circuits, G-causality slightly underestimates the percentage contribution of excitatory connections within the global circuit, but decisively overestimates the percentage contribution of the inhibitory ones. A suggestive possibility, requiring further study, is that inhibitory connectivity is more able to induce a causal link compared with an excitatory one. This provisory result agrees with the observation of several mathematical models, suggesting that inhibitory links are necessary and powerful to synchronize rhythms within a neural circuitry (Campbell & Wang, 1996; Hasenstaub et al., 2005; Ursino et al., 2009).

Finally, a statistical analysis of connectivity conducted using a new brain-inspired circuit (involving both excitatory and inhibitory connections, according to the inhibitory theory of meta-control) further supports the superiority of the conditional estimate with Bonferroni/FDR correction and confirms that G-causality exhibits greater sensitivity to inhibitory versus excitatory connections. In fact, the six inhibitory connections in that circuit are detected in 100% of cases despite the moderate value used within the circuit.

Of course, the present results are representative only of the particular model arrangements, the specific synapse dynamics, and assigned rhythm couplings; hence, they cannot be generalized to any condition. However, they provide important indications on the relationships between G-causality and mechanisms.

### Model Limitations and Future Improvements

Of course, the present results crucially depend on the use of the neural mass model to generate ground truth connections. This model is physiologically sound and has been used in previous papers to simulate brain rhythms (Roe, 2019) and cognitive processes (Bhattacharya et al., 2011; Cona et al., 2014; Cui et al., 2022; Cuppini et al., 2014; Suffczynski et al., 2001; Ursino et al., 2021, 2023; Ursino & Pirazzini, 2024; Wang et al., 2016). As well known, a different category of models makes us of spiking neurons; these models are closer to the natural neuron behavior but are computationally demanding to simulate large brain regions. Future work may repeat a similar analysis on G-causality using more realistic populations of spiking neurons.

A limitation of our model is that it does not reproduce the multilayer arrangement of the cortical column. It is well known that the cortical column is a very organized input/output apparatus; for instance, Layer 4 receives inputs while Layers 2 and 3 provide outputs to other cortical columns (Roe, 2019). This can be represented in future model improvements.

Future studies can also test G-causality in the frequency domain. Of course, rhythm transmission can be better detected in the frequency domain; for instance, the poor theta transmission detected with temporal G-causality could be better detected by looking at a frequency G-causality in the theta band. On the other hand, neural systems are strongly nonlinear, which signifies that intermodulation among frequencies is well-expected, making an analysis in the frequency domain problematic. Future work can examine these advantages and limitations of frequency G-causality.

Recently, G-causality has also been estimated based on nonlinear AR models (Marinazzo et al., 2011; Tank et al., 2022). Due to the impact of nonlinearity in neural dynamics, this may represent a promising future subject of study.

## CONCLUSIONS

In conclusion, our results suggest that G-causality can be used to detect connectivity changes in complex brain networks, and they emphasize the superiority of the conditional versus unconditional estimation in the case of complex nets. The results, however, should be used with caution due to several problems and limitations. First, connectivity changes should be compared using identical conditions (same sampling frequency, model order, signal length, etc.); moreover, the absolute values are not informative per se, but the important information is contained in the relative changes. Second, the absolute value of connectivity depends on the particular rhythm transmitted. Particularly, a small value of G-causality does not necessarily signify the absence of connectivity but indicates a small transmission of information. Third, an important result of our simulation concerns the effect of nonlinearity. As shown in Figure 3, moving the working point of a population toward the upper or lower saturation region drastically reduces the G-causality estimates, confirming that G-causality detects effects, not mechanisms. Hence, the presence of a small G-causality does not necessarily imply the absence of real connectivity but signifies that information is not transmitted from the source region to the target region or that the target region is not significantly affected by the received information.

Finally, G-causality can reveal inhibitory connections more easily than similar excitatory connectivity. The latter distinction (excitation or inhibition) is usually neglected in brain functional connectivity studies.

## METHODS

### The Neural Mass Model

We provide only a qualitative description of the neural mass model here. More details can be found in previous papers by the authors (Pirazzini & Ursino, 2024; Ursino & Pirazzini, 2024) and in the Supporting Information S1.

Each single ROI comprises four neural populations: pyramidal neurons, excitatory interneurons, and inhibitory interneurons with slow and fast synaptic kinetics. Populations are arranged in feedback to simulate the fundamental excitatory and inhibitory connections within a cortical column in a strictly simplified manner. Each population receives an average postsynaptic membrane potential from other neural populations and converts it into average spike density fired by the neurons through a static sigmoidal relationship, thus introducing nonlinearity in neuron behavior (lower threshold and upper saturation).

A second-order dynamics describe each synapse but with different parameters according to the type of population. In particular, three kinds of synapses are implemented: glutamatergic excitatory synapses (from pyramidal neurons and excitatory interneurons), GABAergic inhibitory synapses with slow dynamics (from slow inhibitory interneurons) and with fast dynamics (from fast inhibitory interneurons). Overall, the average numbers of synaptic contacts among neural populations are represented by eight parameters. In addition, an input to the model (a positive mean value + Gaussian white noise), accounting for all exogenous contributions coming from external sources, is provided to pyramidal neurons and fast inhibitory interneurons.

Parameters within each ROI are assigned to simulate a prescribed power spectral density with significant activity in the theta, alpha, beta, or gamma range. Afterward, the different ROIs are connected according to the desired network. The connections among ROIs always start from the pyramidal neurons in the source ROI and reach either pyramidal neurons in the target ROI (thus realizing an excitatory connection) or fast inhibitory interneurons in the target ROI (thus realizing an inhibitory disynaptic connection). The connections between ROIs, both excitatory and inhibitory, are characterized by a strength and a temporal delay.

Simulations were performed using two or more ROIs reciprocally connected. G-causality was computed from one ROI to another ROI using the time series corresponding to the simulated membrane potential of pyramidal neurons of each ROI, representing a good approximation of EEG or mean-field potential for each ROI. The estimated G-causality was plotted against the imposed connectivity strength for comparison.

Since G-causality is more suitable for evaluating effects than mechanisms, we deemed it useful to repeat the previous comparison (in case of models with just two connections) by using the postsynaptic current as the ground truth instead of the synaptic strength (see Supporting Information S1). To this end, we also plotted G-causality versus this current.

Then some parameters were subject to a sensitivity analysis. Among them, one (i.e., the time delay between regions) is internal to the generative model and hence represents a physiological term; the others (AR model order, signal length, sampling frequency) are hyperparameters in the algorithm for G-causality computation. The basal value of these parameters (used in all remaining simulations) was given using a physiological delay (10 ms), the Akaike criterion (Akaike, 1974) as to model order, a sampling frequency equal to twice the Nyquist frequency (after filtering the signals at 50 Hz), and a 10-s signal length.

### G-causality

G-causality was used to estimate functional connectivity between pair of ROIs (pairwise), both in its conditional and unconditional formulation. G-causality is based on an AR modeling framework, enabling the estimation of connection in terms of strength and direction (Granger, 1969).

In detail, given two time series *x*_*i*_(*t*), *x*_*j*_(*t*), representing the activity of *ROI*_*i*_ and *ROI*_*j*_, G-causality quantifies casual interaction from *ROI*_*j*_ to *ROI*_*i*_ (*GC*_*ROI*_*j*_→*ROI*_*i*__) as the extent to which past values of *ROI*_*j*_ improves predictability in *ROI*_*i*_ compared with when the history of *ROI*_*j*_ is excluded.

In its unconditional formulation(pairwise-unconditional G-causality), the restricted AR model contains only the past of *x*_*i*_(*t*) and its compared with the unrestricted AR model containing both the past of *x*_*i*_(*t*) and *x*_*j*_(*t*):

$${GC}_{{ROI}_{j}\to {ROI}_{i}}=\ln\left(\frac{V_{{ROI}_{i}|{ROI}_{i}}}{V_{{ROI}_{i}|{ROI}_{i}{ROI}_{j}}}\right)$$ (1)

where *V*_*ROI*_*i*_∣*ROI*_*i*__ is the variance of the residual for the restricted model, while *V*_*ROI*_*i*_∣*ROI*_*i*_*ROI*_*j*__ is the variance of the residual for the unrestricted model. In this formulation, confounding effects due to common dependencies on signals from other ROIs are not eliminated.

In its conditional formulation (pairwise-conditional G-causality), spurious causalities originating from other signals are removed by introducing these terms into a universe Z of variables in both the restricted and unrestricted models, thus accounting for the joint effect of Z on the inference of *GC*_*ROI*_*j*_→*ROI*_*i*__,

$${GC}_{{ROI}_{j}\to {ROI}_{i}|Z}=\ln\left(\frac{V_{{ROI}_{i}|{ROI}_{i}}|z}{V_{{ROI}_{i}|{ROI}_{i}{ROI}_{j}}|z}\right)$$ (2)

Computations were performed in MATLAB, using functions from two different toolboxes: the BS toolbox, which, in its current version, allows only the computation of the pairwise-unconditional G-causality (Equation 1), and the MVGC toolbox (Barnett & Seth, 2014), which can compute the conditional formulation too (Equation 2) as well as the unconditional one. Variance of each signal was normalized for G-causality computation with both toolboxes.

The basal value of the AR model order was set equal to 15.

### Statistical Inference

Results were also analyzed in terms of the statistical significance of the estimated connectivity against the null hypothesis of null causality, using the *F* test with significance level *α* = 0.05 (Shen & Faraway, 2004; Sureiman & Mangera, 2020).

Moreover, two different kinds of correction were implemented:

1)  Bonferroni: *α*_*new*_ = $\frac{\alpha }{n}$ with *n* number of *p* values being tested. Significant if *p* < *α*_*new*_
2)  False discovery rate via Benjamini-Hochberg procedure (FDR_BH): rank the *p* value in ascending order and then set a cut-off as *th* = $\frac{i}{n}$ * *α* with *n* number of *p* values being tested and *i* the rank of the *p* value in the ordered list. Significant if *p* < *th*

When correction is applied, results are considered statistically significant if the *p* value is below the adjusted significance level (*α*_*new*_ or *th*) of 0.05.

## SUPPORTING INFORMATION

Supporting information for this article is available at https://doi.org/10.1162/NETN.a.38.

## AUTHOR CONTRIBUTIONS

Silvana Pelle: Data curation; Investigation; Methodology; Software; Validation; Visualization; Writing – original draft; Writing – review & editing. Giulia Piermaria: Data curation; Investigation; Methodology; Software; Validation; Visualization; Writing – original draft; Writing – review & editing. Elisa Magosso: Methodology; Supervision; Validation; Writing – original draft; Writing – review & editing. Mauro Ursino: Conceptualization; Data curation; Funding acquisition; Investigation; Methodology; Supervision; Validation; Writing – original draft; Writing – review & editing.

## FUNDING INFORMATION

Research supported by #nextgenerationeu (NextGenerationEU) and funded by the Ministry of University and Research national recovery and resilience plan, project MNESYS (pe0000006)—a multiscale integrated approach to the study of the nervous system in health and disease (dn. 1553 11.10.2022).

## DATA AVAILABILITY STATEMENT

The neural mass model has been implemented in MATLAB. Codes and data will be available on Github. In these codes the authors provide the program for generating the data, an example of the code for unconditional and conditional G-causality computation from data, and instructions on how to replicate each case. Hence, all datasets can be generated from the MATLAB codes.

## Supplementary Material



## TECHNICAL TERMS

Granger causality: A procedure that estimates whether a time series can have a causal influence on another based on its past values.
Brain rhythms: Oscillations in the neuroelectrical activity of neural populations, which can have a strong impact on cognitive processes.
Synchronization: The capacity of signals in different brain parts to remain in unison and to coordinate the reciprocal activities.
Saturation: Maximum response capability of a system, with the output remaining approximately constant for the input above a given limit.
Neural mass: A population of neurons that receive the same inputs and whose activity is strongly synchronized and coordinated.
Brain connectivity: A network of connections among brain areas summarizing the reciprocal effects of the interacting neural populations.
Disynaptic connection: A connection between two neurons that uses two synapses, generally the first excitatory and the second inhibitory.
Excitatory/inhibitory synapses: A connection between two neurons in which the activity of the first contributes to the excitation/ inhibition of the second.
Nonlinearity: A relationship where the superimposition of effects does not hold, hence the sum of different causes produces an abnormal effect.
Conditional estimation: The estimate of a relationship between two signals, which accounts for the influence of other external signals affecting the process.
Power spectral density: An estimate of the amount of power (energy per unit time) contained in the different frequencies of a signal.
Temporal delay: A delay in signal transmission from one part to another due to propagation along an axon (like an electrical cable).
