# Supplementary material for: On the virtues and limitations of Granger-causal brain connectivity estimate: Critical analysis using neural mass models
Source: Netw Neurosci. 2026 Jan 8;10(1):1–24. doi: 10.1162/NETN.a.38 (PMC12798650; doi:10.1162/NETN.a.38)
Supplement: Supplementary file 1 [file netn-10-1-1-s001.pdf]

SUPPLEMENTARY MATERIALS

ON THE VIRTUES AND LIMITATIONS OF GRANGER-CAUSAL BRAIN CONNECTIVITY ESTIMATE: A CRITICAL ANALYSIS USING NEURAL MASS MODELS

Silvana Pelle, Giulia Piermaria, Elisa Magosso, Mauro Ursino*

Department of Electrical, Electronic and Information Engineering Guglielmo Marconi, University of Bologna, Campus of Cesena, I 47521 Cesena, Italy

*Correspondence: mauro.ursino@unibo.it;

Address: Department of Electrical, Electronic, and Information Engineering "Guglielmo Marconi", Area di Campus Cesena, Via Dell'Università 50, I 47521 Cesena FC, Italy

PART 1 - MODEL EQUATIONS AND PARAMETERS

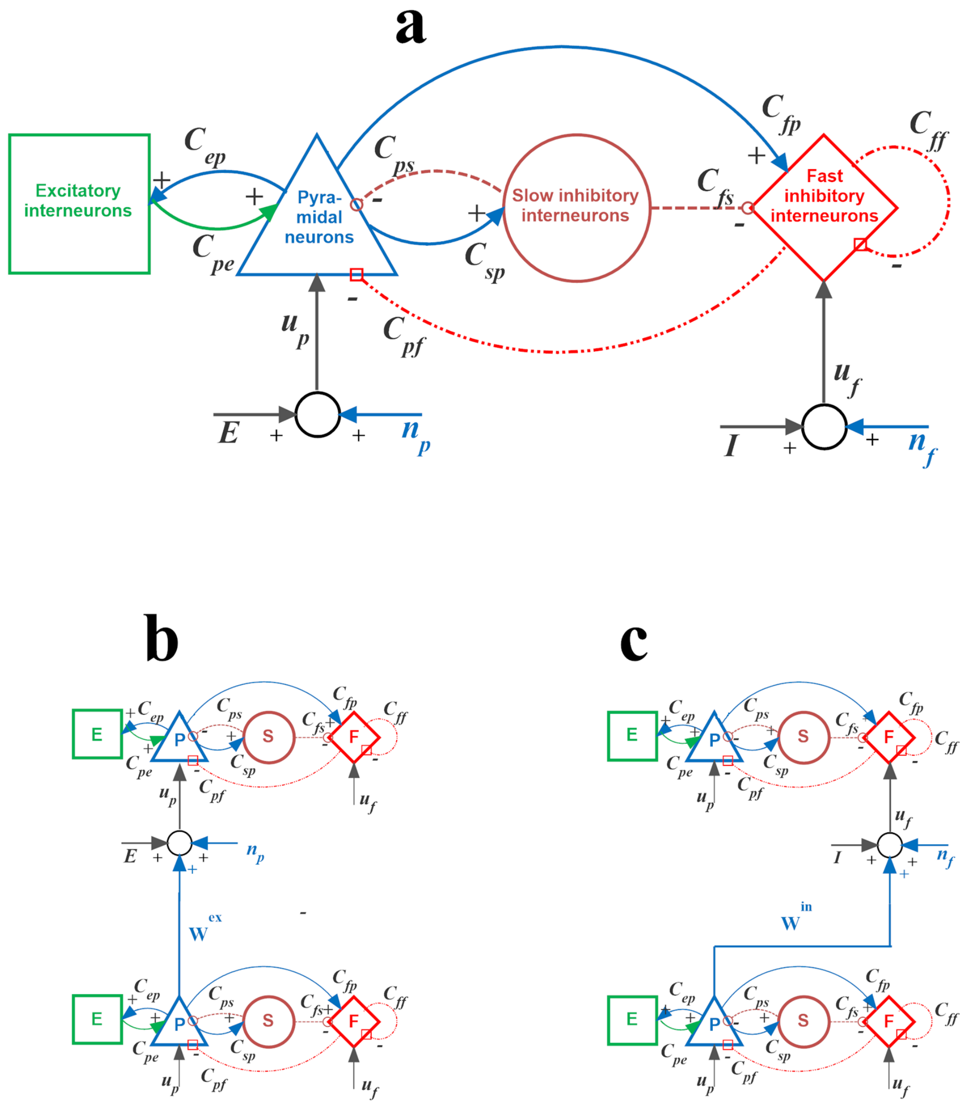

Figure S1 – Upper panel: (a) Neural mass model simulating a single cortical column, the model's single computational unit. The dynamic results from the interaction among a population of pyramidal neurons, one of excitatory interneurons, and two populations of inhibitory interneurons with slow and fast synaptic dynamics, respectively. Continuous blue and green lines represent glutamatergic excitatory synapses. Brown dashed lines are GABAergic inhibitory synapses with slower dynamics, while red dash-dotted lines are GABAergic inhibitory synapses with faster dynamics. The constants C_{ij} represent internal connections among the populations, where the first subscript denotes the target population and the second subscript is the pre-synaptic population. E and I are external inputs to pyramidal neurons and fast inhibitory interneurons, respectively, while n_p and n_f represent white

noise. Bottom panels: two examples of connections among units. The left bottom figure (b) represents an excitatory connection (pyramidal to pyramidal) where W^{ex} is the connection strength. The right bottom figure (c) represents a bi-synaptic inhibitory connection (pyramidal - fast inhibitory – pyramidal) where W^{in} is the connection strength. In these figures, symbols E and I represent the remaining external connections, excluding the one indicated.

Here, we present the model's equations and all related parameters.

Synapses – All synapses in the model are described by the following second order differential equation:

$$\frac{d^2y_n(t)}{dt^2} = \frac{G_n}{\tau_n} z_n(t) - \frac{2}{\tau_n}\frac{dy_n(t)}{dt} - \frac{y_n(t)}{\tau_n^2} \qquad (A1)$$

where G_n is the gain, τ_n is the time constant and z_n is the input to the synapse, i.e., the presynaptic spike density. The subscript n is a generic one; it stands for either p, e, s, or f, depending on the neural population the equation is referring to: p for pyramidal neurons, e for excitatory interneurons, s for slow inhibitory interneurons, f for fast inhibitory interneurons. All second order differential equations of type A1 are equivalent to the two first order differential equations that follow.

$$\begin{cases} \dfrac{dy_n(t)}{dt} = x_n(t) \\ \dfrac{dx_n(t)}{dt} = \dfrac{G_n}{\tau_n} z_n(t) - \dfrac{2}{\tau_n} x_n(t) - \dfrac{y_n(t)}{\tau_n^2} \end{cases} \qquad (A2)$$

Model of a single cortical column – For each neuronal population, we first computed the mean membrane potential *v(t)*, which is influenced by synaptic connections. Then, we computed the average firing rate of the population, *z(t)*, through a sigmoidal activation function, *S(v(t))*. Finally, a normalized post-synaptic potential, *y(t)*, can be computed using the equations A2; the latter must be multiplied by the synaptic weight to determine the actual contribution to the post-synaptic membrane potential.

Both pyramidal neurons and fast inhibitory interneurons can receive an external input – labeled u_p and u_f, respectively. These, when no other interconnected cortical column is incorporated in the model, are random variables n_p and n_f with normal distribution, mean value m_p (or m_f), and standard deviation σ_p (or σ_f). Both represent an average firing rate and reach the target population through an excitatory synapse (blue line in Fig. S1). Specifically, we processed n_p through the excitatory synapse that goes from excitatory interneurons to pyramidal neurons (see Eq. (A4) below), instead of processing the input separately. The other external noise, n_f reaches its target population through a dedicated synapse (see Eq. (A6)).

The membrane potential of pyramidal neurons and fast inhibitory interneurons is also influenced by long-range synapses, which connect different cortical columns. Such contributions are labeled $E(t)$

and $I(t)$ and represent the post-synaptic membrane potential received from the pyramidal neurons of other incorporated cortical columns. They will be discussed in the next paragraph.

Equations for all populations.

Pyramidal neurons :

$$\begin{cases} v_p(t) = C_{pe}y_e(t) - C_{ps}y_s(t) - C_{pf}y_f(t) + E(t) \\ S\big(v_p(t)\big) = z_p(t) = \dfrac{2e_0}{1 + e^{r(s_0 - v_p(t))}} \\ \dfrac{dy_p(t)}{dt} = x_p(t) \\ \dfrac{dx_p(t)}{dt} = \dfrac{G_e}{\tau_e} z_p(t) - \dfrac{2}{\tau_e} x_p(t) - \dfrac{y_p(t)}{\tau_e^2} \end{cases} \quad (A3)$$

Excitatory interneurons:

$$\begin{cases} v_e(t) = C_{ep}y_p(t) \\ S(v_e(t)) = z_e(t) = \dfrac{2e_0}{1 + e^{r(s_0 - v_e(t))}} \\ \dfrac{dy_e(t)}{dt} = x_e(t) \\ \dfrac{dx_e(t)}{dt} = \dfrac{G_e}{\tau_e}\left(z_e(t) + \dfrac{n_p}{C_{pe}}\right) - \dfrac{2}{\tau_e} x_e(t) - \dfrac{y_e(t)}{\tau_e^2} \end{cases} \quad (A4)$$

Slow inhibitory interneurons:

$$\begin{cases} v_s(t) = C_{sp}y_p(t) \\ S(v_s(t)) = z_s(t) = \dfrac{2e_0}{1 + e^{r(s_0 - v_s(t))}} \\ \dfrac{dy_s(t)}{dt} = x_s(t) \\ \dfrac{dx_s(t)}{dt} = \dfrac{G_s}{\tau_s} z_s(t) - \dfrac{2}{\tau_s} x_s(t) - \dfrac{y_s(t)}{\tau_s^2} \end{cases} \quad (A5)$$

Fast inhibitory interneurons:

$$\begin{cases} v_f(t) = C_{fp}y_p(t) - C_{fs}y_s(t) - C_{ff}y_f(t) + y_l(t) + I(t) \\ S(v_f(t)) = z_f(t) = \dfrac{2e_0}{1 + e^{r(s_0 - v_f(t))}} \\ \dfrac{dy_f(t)}{dt} = x_f(t) \\ \dfrac{dx_f(t)}{dt} = \dfrac{G_f}{\tau_f} z_f(t) - \dfrac{2}{\tau_f} x_f(t) - \dfrac{y_f(t)}{\tau_f^2} \\ \dfrac{dy_l(t)}{dt} = x_l(t) \\ \dfrac{dx_l(t)}{dt} = \dfrac{G_e}{\tau_e}(n_f(t)) - \dfrac{2}{\tau_e} x_l(t) - \dfrac{y_l(t)}{\tau_e^2} \end{cases} \qquad (A6)$$

where the subscript l is used to represent the additional synapse, introduced to describe the effect of the noise n_f via glutamatergic dynamics.

Long range connections – As previously mentioned, long-range synapses connect two different regions with different intrinsic rhythms. These contributions appeared in the previous equations as the variables $E(t)$ and $I(t)$ (Eq. (A3) and Eq. (A6)), which stand for long-range Excitation and long-range Inhibition, respectively.

In the following, we will use subscripts i to denote the position of a post-synaptic unit in the model and j for a pre-synaptic unit. $E(t)$ and $I(t)$ are therefore calculated as follows.

Excitation:

$$E_i(t) = \sum_{i=1}^{N} W_{ij}^{ex}\, y_{p,j}(t - D_j) \qquad (A7)$$

Inhibition:

$$I_i(t) = \sum_{i=1}^{N} W_{ij}^{in}\, y_{p,j}(t - D_j) \qquad (A8)$$

where D_j represents the delay in the connectivity N is the number of all presynaptic units, W_{ij}^{ex} is the connection strength of an excitatory connection (pyramidal-pyramidal) from unit j to unit i, and W_{ij}^{in} is the connection strength of a by-synaptic inhibitory connection (pyramidal-fast inhibitory-pyramidal) from unit j to unit i.

The table below lists all parameter values. Note that the noise's variance is divided by the integration step, dt. In this way, we obtain white noise with a power density equal to $\sigma^2\, dt = 5$. The integration step was set to 0.1 ms.

Table S1: Parameters describing the dynamics of the populations within a cortical column:

**Synapses**		**Function S(*v(t)*)**	
Ge (mV)	*5.17*	***e0 (Hz)***	*2.5*
Gs (mV)	*4.45*	***r (mV-1)***	*0.56*
Gf (mV)	*57.1*	***s0 (mV)***	*15*
**Inputs**			
m_f **(Hz)**	0		
σ_p^2 **(s^{-2})**	5/dt		
σ_f^2 **(s^{-2})**	5/dt		

Table: ***Beta rhythm***

**Synapses**		**Intra-column connections**			
τ_e **(ms)**	14.59	C_{ep}	54	C_{fp}	54
τ_s **(ms)**	33.33	C_{pe}	54	C_{fs}	27
τ_f **(ms)**	3.33	C_{sp}	54	C_{pf}	540
**Inputs**		C_{ps}	67.5	C_{ff}	10
m_p **(Hz)**	400				

Table: ***Gamma rhythm***

**Synapses**		**Intra-column connections**			
τ_e **(ms)**	8	C_{ep}	54	C_{fp}	108
τ_s **(ms)**	33.33	C_{pe}	54	C_{fs}	27
τ_f **(ms)**	2.5	C_{sp}	54	C_{pf}	300
**Inputs**		C_{ps}	67.5	C_{ff}	10
m_p **(Hz)**	400				

Table: ***Theta rhythm***

**Synapses**		**Intra-column connections**			
τ_e **(ms)**	13.33	C_{ep}	54	C_{fp}	27
τ_s **(ms)**	33.33	C_{pe}	54	C_{fs}	15
τ_f **(ms)**	3.33	C_{sp}	54	C_{pf}	300
**Inputs**		C_{ps}	67.5	C_{ff}	10
m_p **(Hz)**	400				

Table: ***Alpha rhythm***

**Synapses**		**Intra-column connections**			
τ_e **(ms)**	15.13	C_{ep}	54	C_{fp}	35
τ_s **(ms)**	23.8	C_{pe}	54	C_{fs}	10
τ_f **(ms)**	3.33	C_{sp}	54	C_{pf}	300
**Inputs**		C_{ps}	450	C_{ff}	25
m_p **(Hz)**	250				

The model was used to simulate two or more regions (cortical columns, also named ROIs) reciprocally interconnected via excitatory and/or inhibitory connections. G-causality between a pair of ROIs, using the Brainstorm toolbox (unconditional) and the MVGC toolbox (unconditional and conditional) , was computed on the time series corresponding to the membrane potentials of the pyramidal neurons (v_p) of the simulated regions. For comparison, in the main text, the G-causality estimates from a ROIj to a ROIi were plotted either against the imposed connectivity value (W_{ij}^{ex} or W_{ij}^{in}) or against the total power of the post-synaptic current of that connection i.e. $W_{ij}^{ex} y_{p,j}(t - D_j)$ or $W_{ij}^{in} y_{p,j}(t - D_j)$ (this corresponds to the pre-synaptic input $z_{p,j}$ passed through a glutamatergic synapse providing $y_{p,j}$ and weighted by the synaptic weight).

Moreover, the following parameters were modified in the simulations to assess their effect on G-causality estimation. The parameters subjected to changes were:

- The connectivity strength connecting the ROIs (i.e. W^{ex} or W^{in}), the mean value of the random excitatory input (m_p), the connectivity pure delay (D). These are parameters internal to the generative neural mass model.
- The length of the time series, the AR model order, the sampling frequency of the time series. In particular, the membrane potential time series (obtained at 10 kHz) were subsampled to simulate different sampling rates. These are operative parameters for the algorithm (either Brainstorm or MVGC) computing G-causality.

PART 2 – ADDITIONAL SIMULATIONS AND FIGURES

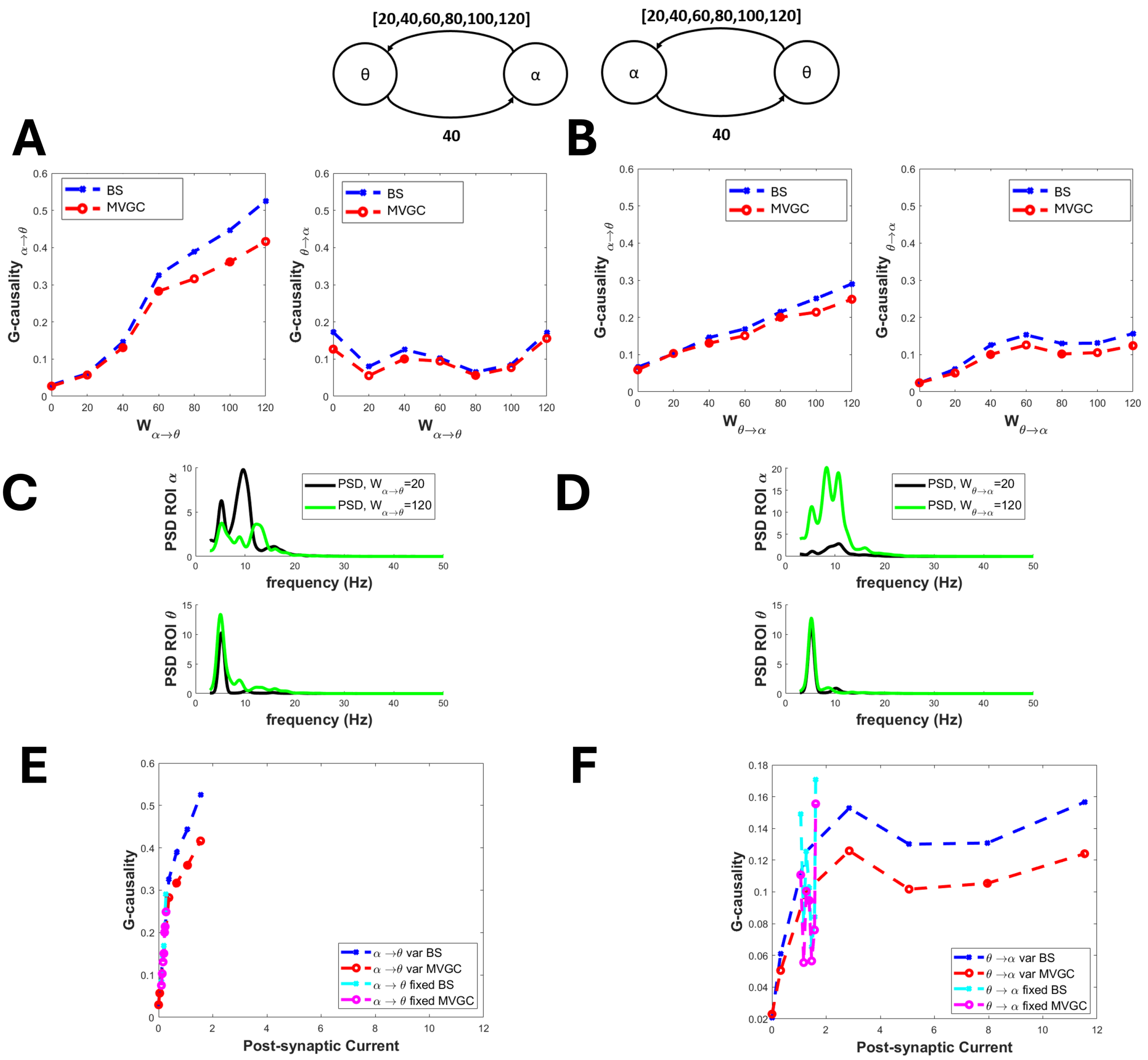

Figure S2 – Effects of a connectivity change on the causal transmission between a theta and an alpha rhythm. G-causality was estimated using both the Brainstorm toolbox (denoted by "BS") and the MVCG toolbox (denoted by "MVGC"). Panels A, B shows that an increase in the connection between alpha and theta regions leads to an increase in the $\alpha \rightarrow \theta$ G-causality, while the reciprocal G-causality remains unbothered. Specifically, for both directions of connectivity, the main changes occur in the coupling from alpha to theta regions. These patterns of connectivity are reflected in the power spectral densities (panels C, D). Panels E, F reflect the relationship between post-synaptic current and GC.

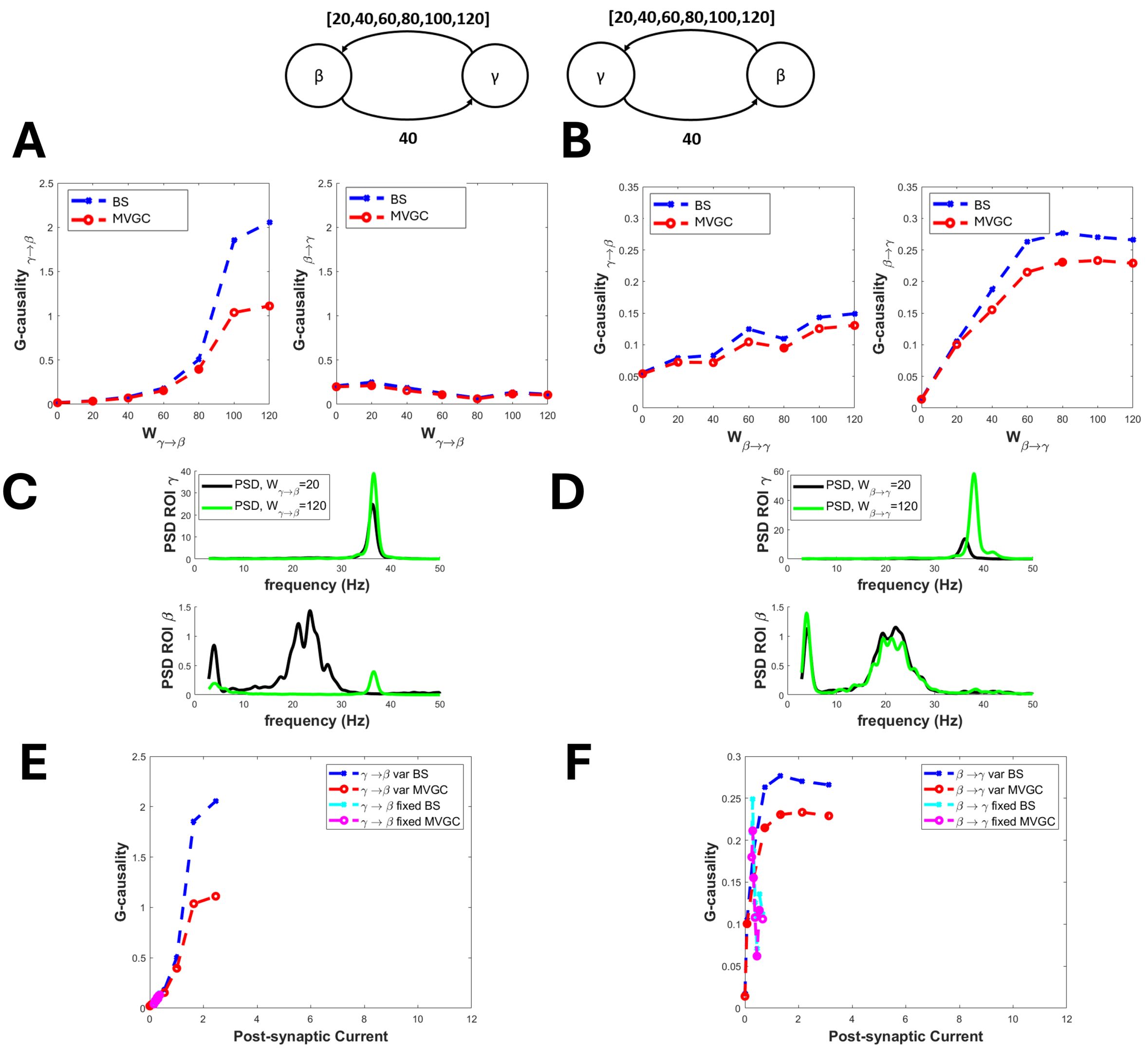

Figure S3 – Effects of a connectivity change on the causal transmission between a beta and a gamma rhythm. G-causality was estimated using both the Brainstorm toolbox (denoted by "BS") and the MVCG toolbox (denoted by "MVGC). G-causality (panel A) exhibits a substantial increase from the gamma region to the beta region when γ → β connectivity increases. Conversely, when the connection from beta to gamma is rising, the G-causality exhibits a slight increase (panel B). Regarding the power spectral density (panels C, D), an increase in the γ → β connectivity leads to a significant power increase in the frequency band 30-40 Hz in the β region. An increase in the β → γ connectivity results in a power increase in the gamma between 30 and 40 Hz. Panels E, F represent the relationship between post-synaptic current and G-causality.

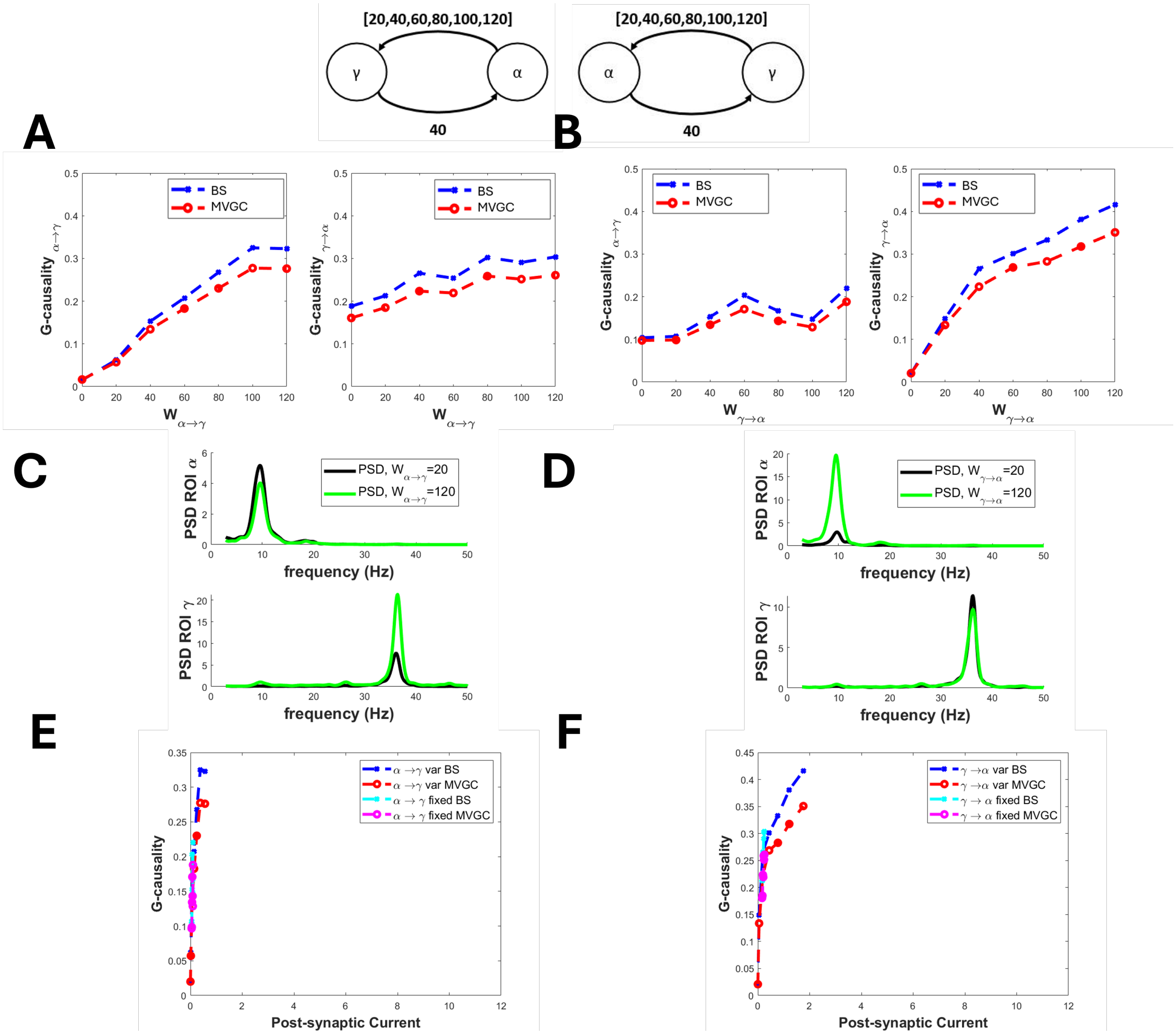

Figure S4 – Effects of a connectivity change on the causal transmission between an alpha and a gamma rhythm. G-causality was estimated using both the Brainstorm toolbox (denoted by "BS") and the MVCG toolbox (denoted by "MVGC"). An increase in the connection from the alpha region to the gamma region results in a smooth increase in alpha-gamma G-causality(panel A). Similarly, an increase in $\gamma \rightarrow \alpha$ connectivity leads to a comparable increase in $\gamma \rightarrow \alpha$ G-causality (panel B). The power spectral density (panel C, D) reveals that an increase in $\alpha \rightarrow \gamma$ connectivity enhances the spectrum in the gamma band and introduces alpha power in the gamma region. Conversely, an increase in the connectivity $\gamma \rightarrow \alpha$ amplifies the power in the alpha region with only minor changes

in the gamma one. Panels E, F represent the relationship between post-synaptic current and G-causality.

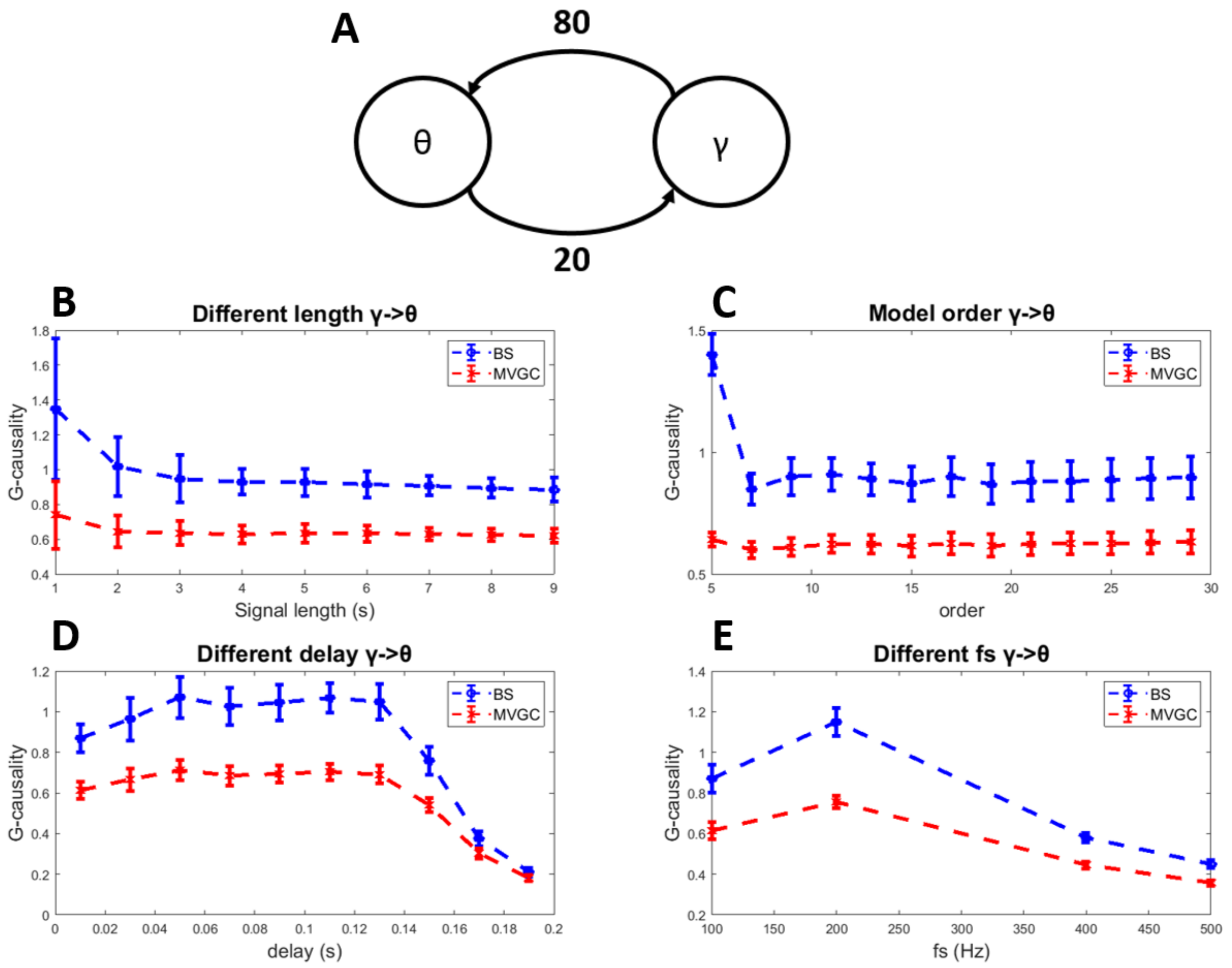

Figure S5 – Dependence of G-causality on signal length, AR model order, connectivity pure delay, and sampling frequency, realized assuming two regions interconnected with reciprocal excitatory synapses as in the upper panel ($\theta - \gamma$ reciprocal connection). G-causality was estimated using both the Brainstorm toolbox (blue lines denoted by "BS") and the MVCG toolbox (red lines denoted by "MVGC"). Results are provided as mean+std over 20 trials. Panel B reports the change in G-causality due to different signal lengths: signal length should be at least 2-3 s. Panel C depicts the effect of the AR model order on G-causality estimation: suitable values are more than 10. Panel D reports the effect of pure delay: estimation degrades over 150 ms of connectivity delay. Panel E shows the effect

of sampling frequency: at high sampling frequencies, the estimated G-causality decreases, with a low standard deviation.

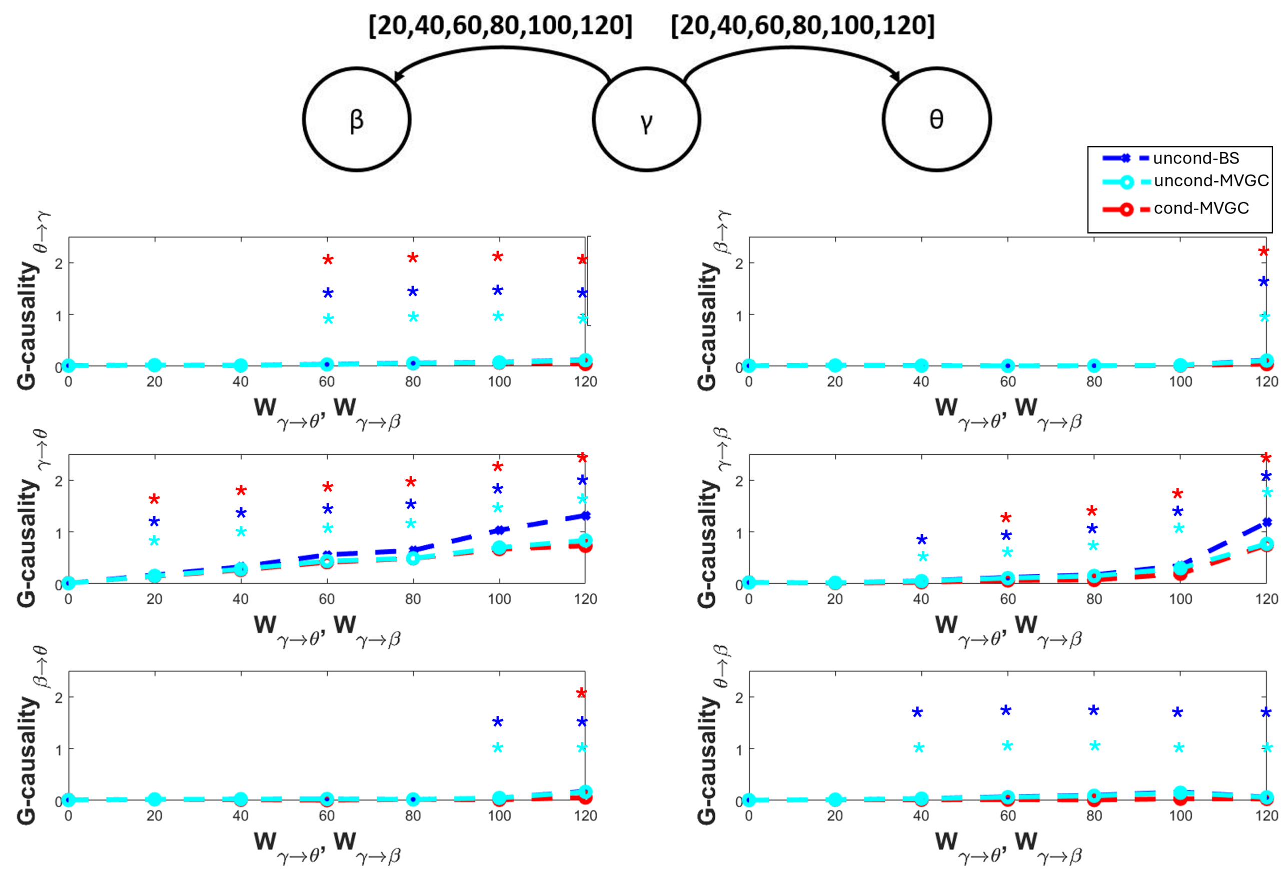

Figure S6 – Effect of a common input on G-causality estimation. Specifically, the common input originates from the gamma region and enters the beta and theta regions with synaptic strength ranging from 20 to 120. The other panels represent the G-causality estimate of all six possible connections. G-causality was estimated using both the unconditional Brainstorm toolbox (blue lines denoted by "uncond-BS") and the MVCG toolbox (unconditional formulation, cyan lines denoted with "uncond-MVGC", and conditional formulation, red lines denoted by "cond-MVGC"). Asterisks denote statistically significant G-causality at 5% (blue, cyan, or red, as in the corresponding plots). G-causality $\gamma \rightarrow \theta$ and $\gamma \rightarrow \beta$ (corresponding to true synaptic connections) is statistically significant for synaptic strength higher or equal to 40. Interestingly, a feedback connection from $\theta \rightarrow \gamma$ theta to gamma is found when $W_{\theta\rightarrow\gamma}$, $W_{\beta\rightarrow\gamma} \geq 60$. "False connectivity" $\beta \rightarrow \theta$ and $\theta \rightarrow \beta$, caused by the common input, are especially detected by the unconditional estimators, whereas the conditional estimator is more robust against these spurious effects.

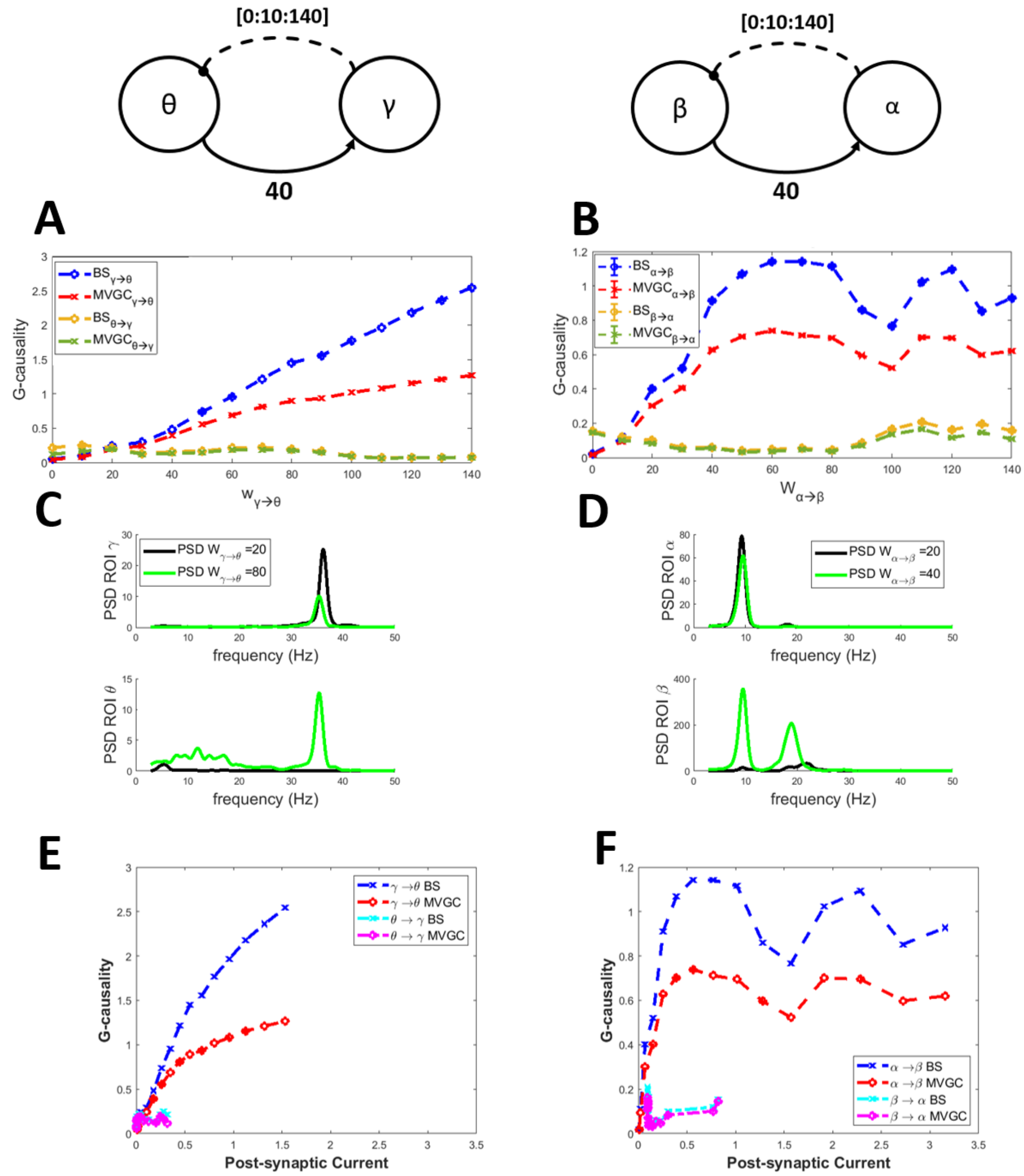

Figure S7– Effect of the change of an inhibitory connection on G-causality estimate. The simulations were performed using two regions with different rhythms (θ – γ: left panels, or α – β: right panels) coupled via one inhibitory and one excitatory connection. Panels A, B refer to a progressive increase in the inhibitory connection (from 0 to 140), while the excitatory connection is kept constant. G-causality was estimated using both the Brainstorm toolbox (blue and brown lines denote by “BS”) and the multivariate MVCG toolbox (red and green lines denoted by “MVCG”). It is worth noting that the effect of inhibitory synapses on G-causality is stronger than the effect of excitatory synapses. Panels C, D show the power spectral densities. The presence of a strong inhibitory connection is able

to produce a clear rhythm in the target region. Panels E, F show the relationships of G-causality vs. post-synaptic current in all cases.
